# Characterization of design grammar of peptides for regulating liquid droplets and aggregates of FUS

**DOI:** 10.1038/s41598-021-86098-1

**Published:** 2021-03-23

**Authors:** Kiyoto Kamagata, Rika Chiba, Ichiro Kawahata, Nanako Iwaki, Saori Kanbayashi, Kana Maeda, Hiroto Takahashi, Atsushi Hirano, Koji Fukunaga, Keisuke Ikeda, Tomoshi Kameda

**Affiliations:** 1 Institute of Multidisciplinary Research for Advanced Materials, Tohoku University, Katahira 2-1-1, Aoba-ku, Sendai, 980-8577 Japan; 2 Graduate School of Life Sciences, Tohoku University, Sendai, 980-8577 Japan; 3 Department of Chemistry, Graduate School of Science, Tohoku University, Sendai, 980-8578 Japan; 4 Department of Pharmacology, Graduate School of Pharmaceutical Sciences, Tohoku University, Sendai, 980-8578 Japan; 5 Nanomaterials Research Institute, National Institute of Advanced Industrial Science and Technology (AIST), Tsukuba, Ibaraki 305-8565 Japan; 6 Department of Biointerface Chemistry, Faculty of Pharmaceutical Sciences, University of Toyama, 2630 Sugitani, Toyama, 930-0194 Japan; 7 Artificial Intelligence Research Center, National Institute of Advanced Industrial Science and Technology (AIST), Koto, Tokyo 135-0064 Japan

**Keywords:** Peptides, Biophysical chemistry, Intrinsically disordered proteins

## Abstract

Liquid droplets of aggregation-prone proteins, which become hydrogels or form amyloid fibrils, are a potential target for drug discovery. In this study, we proposed an experiment-guided protocol for characterizing the design grammar of peptides that can regulate droplet formation and aggregation. The protocol essentially involves investigation of 19 amino acid additives and polymerization of the identified amino acids. As a proof of concept, we applied this protocol to fused in sarcoma (FUS). First, we evaluated 19 amino acid additives for an FUS solution and identified Arg and Tyr as suppressors of droplet formation. Molecular dynamics simulations suggested that the Arg additive interacts with specific residues of FUS, thereby inhibiting the cation–π and electrostatic interactions between the FUS molecules. Second, we observed that Arg polymers promote FUS droplet formation, unlike Arg monomers, by bridging the FUS molecules. Third, we found that the Arg additive suppressed solid aggregate formation of FUS, while Arg polymer enhanced it. Finally, we observed that amyloid-forming peptides induced the conversion of FUS droplets to solid aggregates of FUS. The developed protocol could be used for the primary design of peptides controlling liquid droplets and aggregates of proteins.

## Introduction

Liquid–liquid phase separation (LLPS) of proteins results in the formation of a condensed phase known as liquid droplets in a dilute bulk phase, which enables a wide variety of cellular functions and their regulation at levels that cannot be achieved by the dilute bulk phase alone. Droplet formation is involved in processes such as transcription, condensation of DNA, and DNA repair. However, liquid droplets are associated with a potential risk of becoming solid aggregates or forming amyloid fibers, which can cause diseases. Fused in sarcoma (FUS), a model protein used in this study, forms liquid droplets to perform biological functions; however, the conversion of FUS to hydrogel and amyloid fibers causes neurodegenerative diseases^1–5^. Aggregation-associated diseases can develop because of failure to maintain liquid droplet homeostasis. Accordingly, the liquid droplets of aggregation-prone proteins are a potential target for drug discovery.

The RNA-binding protein FUS participates in RNA transcription, splicing, transport, and translation. FUS consists of an N-terminal SYGQ-rich low-complexity (LC) domain and a C-terminal RNA binding domain containing three RGG-rich domains: an RNA recognition motif (RRM) domain, a zinc finger (ZnF) domain, and a nuclear localization signal (NLS) domain. The RRM and zinc finger domains have a small globular fold, whereas the other regions are intrinsically disordered. FUS undergoes phase separation among the dilute bulk phase, liquid droplet state (spherical cluster with high dynamics), hydrogel state (spherical cluster with limited dynamics), and solid aggregation state including amyloid fiber (non-spherical cluster). The phase separation of FUS is primary driven by self-association of LC domains via cross-β structures^3,6,7^ and regulated by post-translational modifications^7,8^. The liquid droplets are also mediated by multivalent cation–π interactions among Arg residues of the RNA-binding domain and Tyr residues of the LC domain^9,10^. Additionally, Lys and Phe participate in droplet formation via the weak cation–π interactions^9^. Furthermore, other types of interactions are involved: electrostatic interactions with Asp^9^; π–π interactions between Tyr and Phe^9^; intermolecular β-sheet hydrogen bonding^10^; and hydrogen bonding, π–sp^2^ interactions, and/or hydrophobic interactions with Gln in the LC domain^11^. The structure in the disordered regions of the droplet is controversial; any secondary structure is not induced^11,12^, while cross-β structure is present^7,13^. The liquid droplets can transform into hydrogels^3,7,10,14^ and amyloid fibrils^4,6,7,13,15^. The 37th–94th residues in the LC domain form the fibril core^7,13^.

Despite extensive investigations on the molecular interactions in phase separations of FUS, studies on phase-separation regulators were limited to several endogenous molecules, except for 1,6-hexanediol. ATP promotes liquid-droplet formation at a low concentration but suppresses it at a high concentration^16,17^. RNA, 1,6-Hexanediol, nuclear import receptor, and small heat-shock protein 27 suppresses droplet formation^18–21^. Ubiquitin 2 modulates the LLPS of the FUS–RNA complex^22^.

Unlike these endogenous molecules, artificial peptides can potentially target disease-associated LLPS proteins and serve as promising drug candidates. High affinity of the peptides for the target disordered region of the proteins is attributed to their flexible fitting to any conformation of the disordered regions based on the combination of the 20 amino acids with different characteristics. A peptide was designed that succeeded in targeting the disordered region of p53 and modulating its function^23^. Additionally, a short amyloid-genic peptide from FUS was observed to induce the aggregation of the LC domain of FUS^15^. Considering these facts, peptide design is a promising approach for regulating the LLPS behavior. However, the design grammar for the peptides remains unclear. In addition, the theoretical pool of peptides is considerably large; for example, 20^10^ candidates are possible for a 10-residue peptide. An efficient search strategy needs to be developed for identifying LLPS-regulating peptides.

In this study, we proposed an experiment-guided protocol for characterizing the design grammar of peptides that can regulate droplet formation and aggregation. The protocol essentially involves investigation of 19 amino acid additives and polymerization of the identified amino acids. As a proof of concept, we demonstrated the effect of amino acids and peptides on formation of the droplets and solid aggregates of the model protein FUS. We further determined the molecular mechanism underlying this process using MD simulations.

## Results

### Characterization of MBP-tagged FUS used in the droplet-formation study

**Figure 1:**
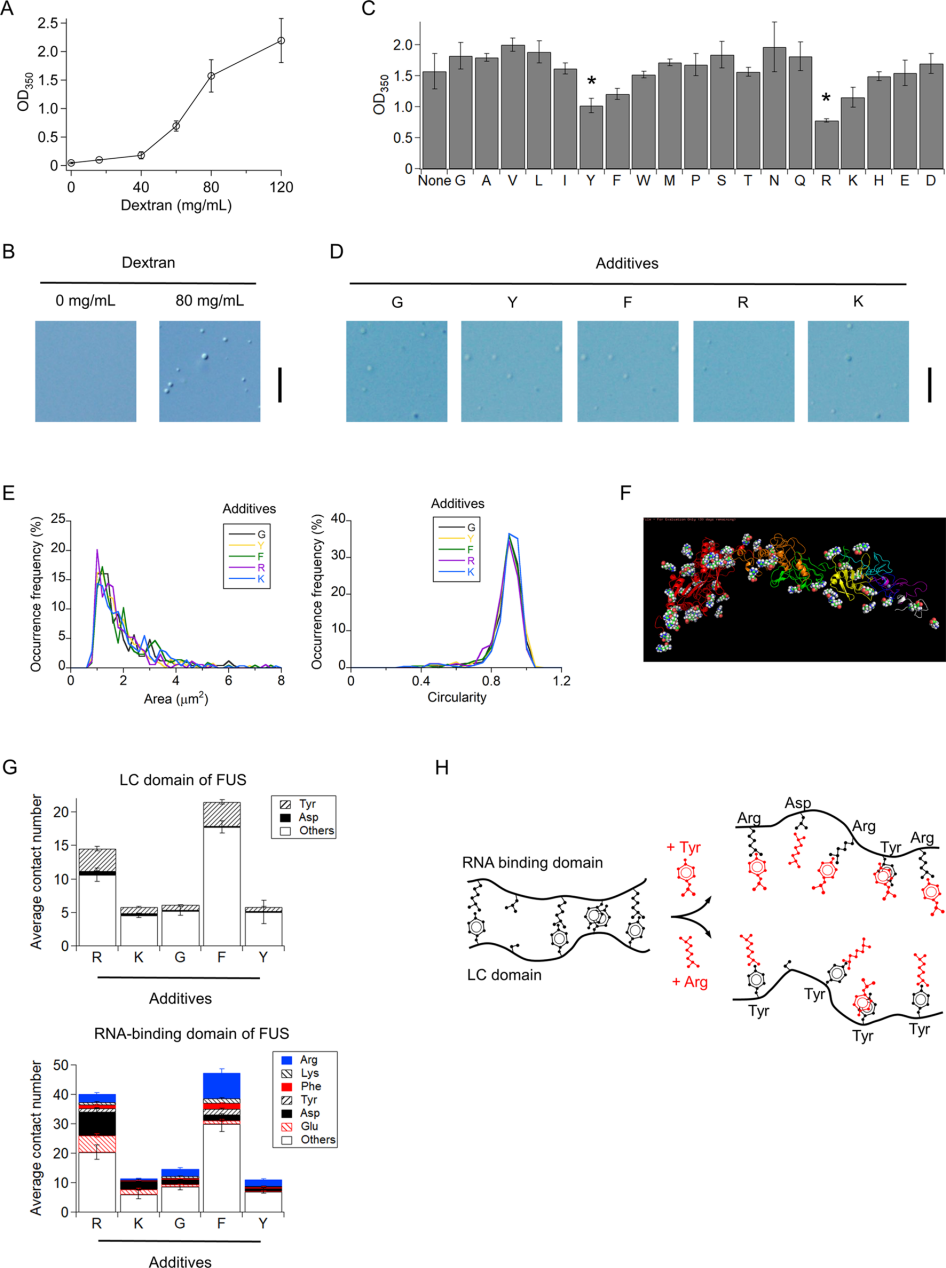
Arg and Tyr additives suppress the formation of FUS droplets. (**A**) Dependence of MBP-FUS droplet formation on dextran concentration was detected as scattering at 350 nm (OD_350_). (**B**) DIC images of MBP-FUS solutions in the presence/absence of 80 mg/mL dextran. (**C**) Effect of 19 amino acids as additives for MBP-FUS droplet formation detected as OD_350_. "None" denotes the absence of additives. The solution contained 80 mg/mL dextran and 40 mM amino acids, except for Tyr or 2 mM Tyr owing to low solubility. Asterisks indicate a significant difference (*P* = 0.044 for Tyr and Gly and 0.039 for Arg and Gly, two-tailed *t* test). (**D**) DIC images of MBP-FUS solutions in the presence of typical amino acids. (**E**) Distributions of cross-section area and circularity of individual droplets of MBP-FUS in the presence of typical amino acids; 177–293 droplets were analyzed. (**F**) A typical snapshot of MBP-FUS interacting with Arg additives in MD simulation. Red, orange, green, yellow, light blue, blue, purple, and white ribbon structures denote the MBP, LC, RGG1, RRM, RGG2, ZnF, RGG3, and PY-NLS domains of FUS, respectively. Space-filled structures represent Arg additives that form contacts with MBP-FUS. (**G**) Average number of contacts between the amino acid additives and intact residues of the LC domain (top) and RNA-binding domain (bottom) of FUS in the molecular dynamics simulations. RNA-binding domain includes RGG1, RRM, RGG2, ZnF, RGG3, and PY-NLS domains. The residues of FUS are classified into droplet-related residues plus Glu (colors or shaded) or others (blank). (**H**) Schematic diagram of competitive suppression of FUS droplet by Arg and Tyr additives. Error bars in (**A**, **C**) represent the standard errors (*N* ≥ 3). Scale bars in (**B**, **D**) denote 20 µm and 10 µm, respectively.

Because FUS is an aggregation-prone protein, an N-terminal of FUS is commonly conjugated with a solubility tag—maltose-binding protein (MBP) tag—for storage^8,20^. This tag is generally cleaved using tobacco etch virus (TEV) protease to induce FUS droplet formation. However, we used MBP-tagged FUS (MBP-FUS) without cleavage for most parts of this study to simplify the system; if the tag-cleavage step was used, the system could contain FUS, MBP-FUS, and MBP, as well as TEV protease. In this study, a molecular clowder dextran was added for inducing droplet formation, instead of the MBP cleavage. When the dextran concentration was higher than 60 mg/mL, we detected an increase in light scattering from 5 µM MBP-FUS solution at 350 nm (Fig. 1A). Imaging of the solutions using a differential interference contrast (DIC) microscope identified micrometer-sized spherical droplets in the presence of 80 mg/mL dextran (Fig. 1B). These results are consistent with the crowding-enhanced droplet formation observed for non-tagged FUS^9^. The droplets were formed through incubation for 10 min at 20 °C after adding the MBP-FUS stock solution into the dextran solution. We conducted subsequent measurements for the FUS droplets in the presence of 80 mg/mL dextran, except for MBP-tag cleavage experiments. As a control, MBP tag without FUS did not form droplets in 80 mg/mL dextran (Supplementary Fig. S1).

### Arg and Tyr additives suppressed FUS droplet formation

We examined the effect of 19 amino acid additives on the formation of MBP-FUS droplets. The amino acids were used at 40 mM except for Tyr, which used at 2 mM, because of the low solubility of Tyr. Cys was not tested, because Cys can form disulfide bonds to the Cys residues of proteins in general. The addition of Gly did not alter the scattering intensity from the MBP-FUS solution (Fig. 1C). Similarly, the other amino acids, except for Arg, Lys, Tyr, and Phe, did not affect droplet formation (Fig. 1C). In contrast, Arg and Tyr significantly decreased the scattering intensity (*p* = 0.044 for Tyr and Gly and 0.039 for Arg and Gly, two-tailed t-test). Arg suppressed droplet formation at concentrations greater than 5 mM, and the scattering intensity from the droplet was saturated at 40 mM Arg (Supplementary Fig. S2A). For Lys or Phe, the scattering was reduced to a less extent (*p* = 0.063 for Lys and 0.074 for Phe, two-tailed *t*-test with Gly). Accordingly, the four amino acids suppress the formation of MBP-FUS droplets. Next, we examined the effect of the amino acids on the size and shape of the droplets using DIC microscopy. Spherical droplets of MBP-FUS were observed in the presence of any of the 19 amino acids (Fig. 1D and supplementary Fig. S3). Arg, Lys, Tyr, and Phe did not affect the relative distribution of the cross-section area and circularity of droplets compared to Gly as the control (Fig. 1E).

We examined how such effective additives suppress the formation of MBP-FUS droplets using MD simulations. Molecular dynamics of MBP-FUS was simulated in the presence of Arg, Lys, Phe, or Gly at 100 mM or Tyr at 10 mM. In the simulations, MBP-FUS exhibited a rod-like shape, consistent with that observed through small-angle X-ray scattering analysis^20^ (Fig. 1F and Supplementary Fig. S4). We analyzed the number of contacts between the additives and residues of MBP-FUS, where the contact was defined as a distance of less than 6.5 Å between the centers of two side chains, except for Gly, for which Cα was used (Fig. 1G). We focused on five residues that participate in droplet formation (Arg, Lys, Phe, Tyr, and Asp)^9^ and Glu with a negative charge. In particular, the Arg additive exhibited more contacts with Tyr residues of the LC domain and Asp/Glu residues of the RNA-binding domain (Asp residues of the RGG1 domain and Asp/Glu residues of the RRM domain, Supplementary Fig. S5A) than the Gly additive. The cation–π, π–π, and electrostatic interactions between the Arg additives and FUS molecules inhibit the interactions among the FUS molecules. In contrast, the Tyr additive formed contacts with Arg residues of the RNA-binding domain of FUS via cation–π interaction. Accordingly, we propose that Arg and Tyr interact with the droplet-forming residues of FUS, thereby competitively inhibiting the interactions among the FUS molecules and suppressing droplet formation (Fig. 1H).

### Poly-Arg additive enhanced FUS droplet formation by bridging FUS molecules

**Figure 2:**
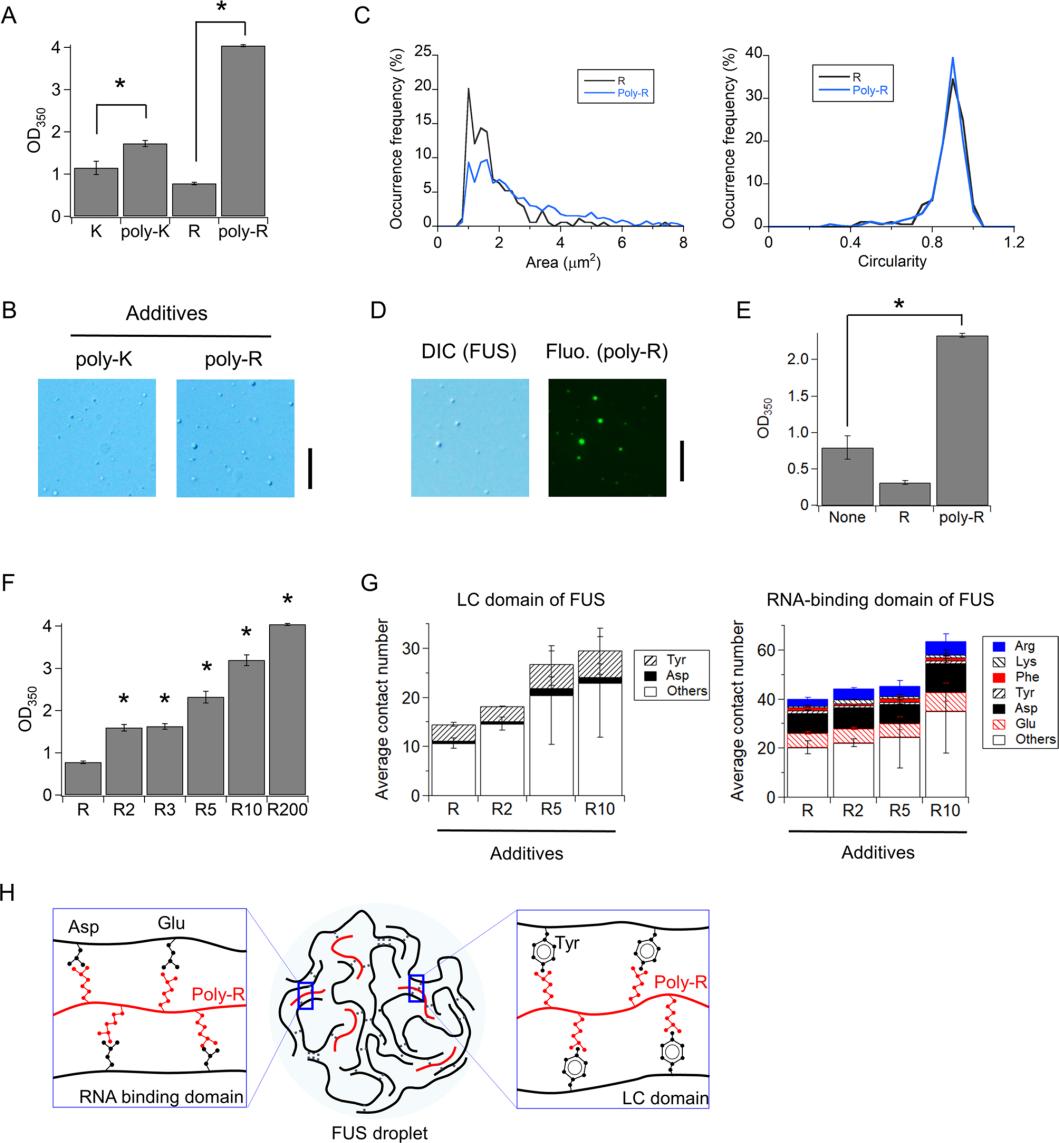
Polymerization of Arg and Lys promotes the formation of FUS droplets. (**A**) Effect of polymerization of Arg and Lys on MBP-FUS droplet formation detected as the scattering at 350 nm. Arg and Lys concentrations in monomer were set to 40 mM for monomers and polymers. Poly-Lys and poly-Arg are composed of 220-mer and 200-mer in median, respectively. Error bars represent the standard errors (*N* = 3). Asterisks indicate a significant difference (*p* = 0.045 for poly-Lys and < 0.0001 for poly-Arg (R200), two-tailed t-test). (**B**) DIC images of MBP-FUS solutions in the presence of Lys/Arg monomer and polymer. Scale bar denotes 20 µm. (**C**) Distributions of cross-section area and circularity of individual droplets of MBP-FUS in the presence of Arg monomer (*N* = 177) and polymer (*N* = 829). D) DIC and fluorescence microscopic images of MBP-FUS at 5 µM and Alexa488-labeled poly-R at 0.1 µM. (**E**) Effect of MBP tag removal on FUS droplet formation detected as the scattering at 350 nm in the absence of Dextran. Error bars represent the standard errors (*N* = 3). Asterisk indicates a significant difference (*p* = 0.01 for poly-Arg, two-tailed t-test). "None" denotes the absence of additives. F) Effect of polymer length of Arg to MBP-FUS droplet formation detected as OD_350_. Arg concentrations in monomer were set to 40 mM for monomer and polymers: 20 mM R2, 13 mM R3, and 4 mM R10. Error bars represent the standard errors (*N* = 3 except for R2 and *N* = 6 for R2). The data for R200 was the same as that for poly-R in (**A**). Asterisks indicate a significant difference between monomer and polymers (*p* < 0.0001 for R2, *p* = 0.019 for R3, *p* = 0.008 for R5, and *p* = 0.008 for R10; two-tailed t-test). (**G**) Molecular dynamics simulations of MBP-FUS in the presence of R, R2, R5, and R10 additives. The left and right panels show the average number of contacts between the additives and intact residues of LC- and RNA-binding domains of FUS, respectively. The colored or shaded and blank intact residues denote droplet-related residues plus Glu and others, respectively. (**H**) Schematic diagram of FUS droplets promoted by Arg polymer. Arg polymer connects intact Tyr, Asp, and Glu residues of different FUS molecules, thus promoting FUS droplets. In (**A**–**D**, **F**), the solutions contained 80 mg/mL dextran.

We hypothesized that, compared with Arg monomers, Arg polymers interact more effectively with FUS, because Arg polymers have the potential to form multivalent interactions. To test this hypothesis, we investigated the effect of Arg polymers on the formation of MBP-FUS droplets. Poly-Arg (15–70 kDa; median, 200-mer; R200) significantly enhanced the scattering intensity from MBP-FUS droplets, at a concentration of 40 mM with respect to Arg units (*p* < 0.0001, two-tailed *t*-test, Fig. 2A); in contrast, Arg monomer suppressed droplet formation. Poly-Arg effectively promoted droplet formation at concentrations greater than 0.5 mM with respect to Arg units (Supplementary Fig. S2B). Additionally, poly-Lys (30–70 kDa; median, 220-mer) increased the scattering intensity compared to Lys monomer (*p* = 0.045, two-tailed *t*-test, Fig. 2A). The effect of poly-Lys was lesser than that of poly-Arg likely due to the weaker cation–π and electrostatic interactions with FUS (Fig. 1F). The DIC images displayed the MBP-FUS droplets in the presence of poly-Arg (Fig. 2B). The occurrence percentage of large droplets with cross-section area greater than 3 µm^2^ was increased, coincident with the decrease in small droplets with 1–2 µm^2^ (Fig. 2C). The circular shape of the droplets was not considerably changed (Fig. 2C). In contrast, poly-Lys did not affect the shape and size of the droplets, supporting the observation that the poly-Lys was less effective in regulating droplet formation than poly-Arg (Supplementary Fig. S6). We additionally confirmed the uptake of poly-Arg labeled with a fluorophore, Alexa488, into the MBP-FUS droplets using DIC and fluorescence microscopy (Fig. 2D). Furthermore, we confirmed the effect of poly-Arg on the formation of FUS droplets following the cleavage of the MBP tag by TEV protease in the absence of dextran (*p* = 0.01, two-tailed *t*-test, Fig. 2E). Overall, the results demonstrated that the polymer of Arg significantly promoted the FUS droplet formation, while Arg monomers exhibited the opposite effect.

To identify the effective peptide length for promoting FUS droplet formation, we examined the effect of Arg peptide length on the formation of MBP-FUS droplets. The peptide concentrations were adjusted to 40 mM in the Arg monomer unit. The scattering intensities of Arg dimer (R2) and trimer (R3) were higher than that of the Arg monomer (*p* < 0.02, two-tailed *t*-test, Fig. 2F), comparable to that in the absence of additives (Fig. 1C). In 5-mer peptide (R5), the intensity was further enhanced (*p* = 0.008, two-tailed *t*-test, Fig. 2F). The MD simulations of MBP-FUS in the presence of R, R2, R5 or R10 showed similar patterns, that is, an increase in the absolute number of contacts in the LC- and RNA-binding domains with increase in the Arg peptide length (Fig. 2G). Additionally, the polymerization of Arg did not affect the binding regions over the FUS sequence (Supplementary Figs. S5B and S7). Collectively, the polymer length of Arg affects the droplet formation tendency, likely because the more Arg residues the polymers have, the more strongly they interact with FUS molecules via cation–π and electrostatic interactions, leading to FUS bridging and hence FUS droplet formation (Fig. 2H).

### Arg and poly-Arg additives regulated FUS cluster formation similarly in cells

**Figure 3:**
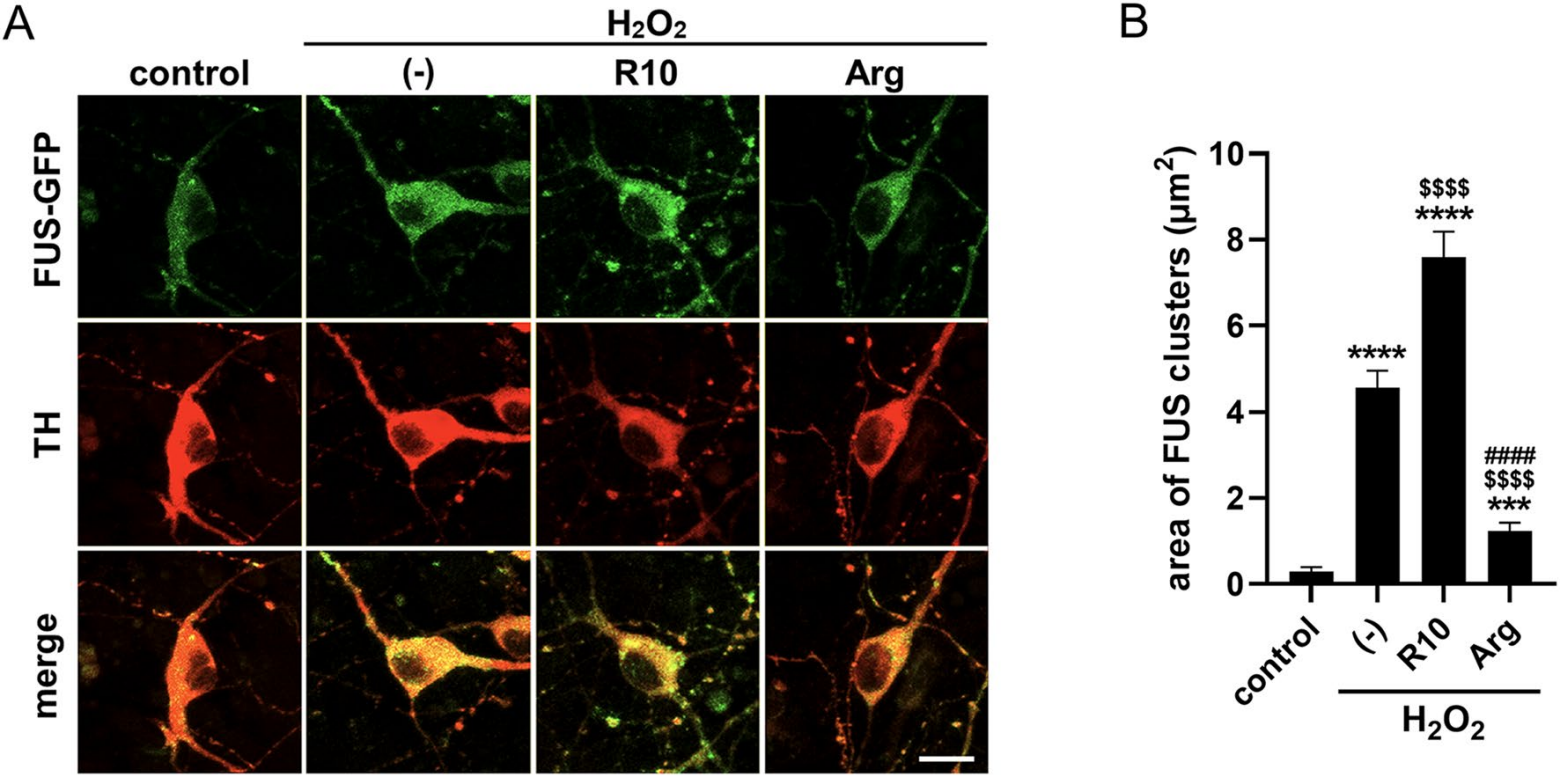
Arg and poly-arg additives worked similarly for regulating FUS cluster formation in cultured dopaminergic neurons. (**A**) Effect of Arg and poly-Arg additives to FUS cluster formation in the cells detected by a confocal laser scanning microscopy. Cells were transfected with FUS-GFP and exposed to H_2_O_2_ with no additive ( −), R10, or Arg. FUS-GFP (green) and anti-tyrosine hydroxylase (TH) antibody (neuron cell marker, red) were respectively detected. Scale bar denotes 10 µm. (**B**) Quantification of the area of FUS clusters in TH^+^ neurons. Error bars represent the standard errors (*N* = 20). *** and **** indicate significant differences (*p* < 0.001 and *p* < 0.0001 for control) in one-way analysis of variance (ANOVA) with post-hoc Tukey’s multiple comparison test, respectively. $$$$ and #### indicate significant differences (*p* < 0.0001 for no additive ( −) and *p* < 0.0001 for R10) in one-way ANOVA with post-hoc Tukey’s multiple comparison test, respectively.

To test if Arg and poly-Arg additives work for regulating FUS clusters formed in cells, we expressed FUS-GFP in dopaminergic neurons and examined the effect of these additives (Fig. 3A). H_2_O_2_ treatment was used for triggering the formation of FUS clusters. In fact, the clusters of FUS-GFP (corresponding to yellow or green circles in the merge images of Fig. 3A) were increased by H_2_O_2_ treatment (control vs H_2_O_2_ with no additive in Fig. 3A). The average area of FUS clusters upon H_2_O_2_ treatment, calculated from the images, increased 15-fold (Fig. 3B). When R10 was added in H_2_O_2_, the average area of FUS clusters in neurons were further increased 1.7-fold (Fig. 3A,B). We assumed the action by R10 inside the cell, since R9 and other arginine-rich peptides are known as a cell-penetrating peptide^24^. In contrast, the addition of Arg decreased the area of FUS clusters significantly (Fig. 3A,B). Therefore, these results demonstrated that Arg and poly-Arg additives can work for regulating FUS cluster formation in cells in a way similar to in vitro conditions.

### Arg additives suppressed solid aggregate formation of FUS, but poly-Arg additive enhanced it

**Figure 4:**
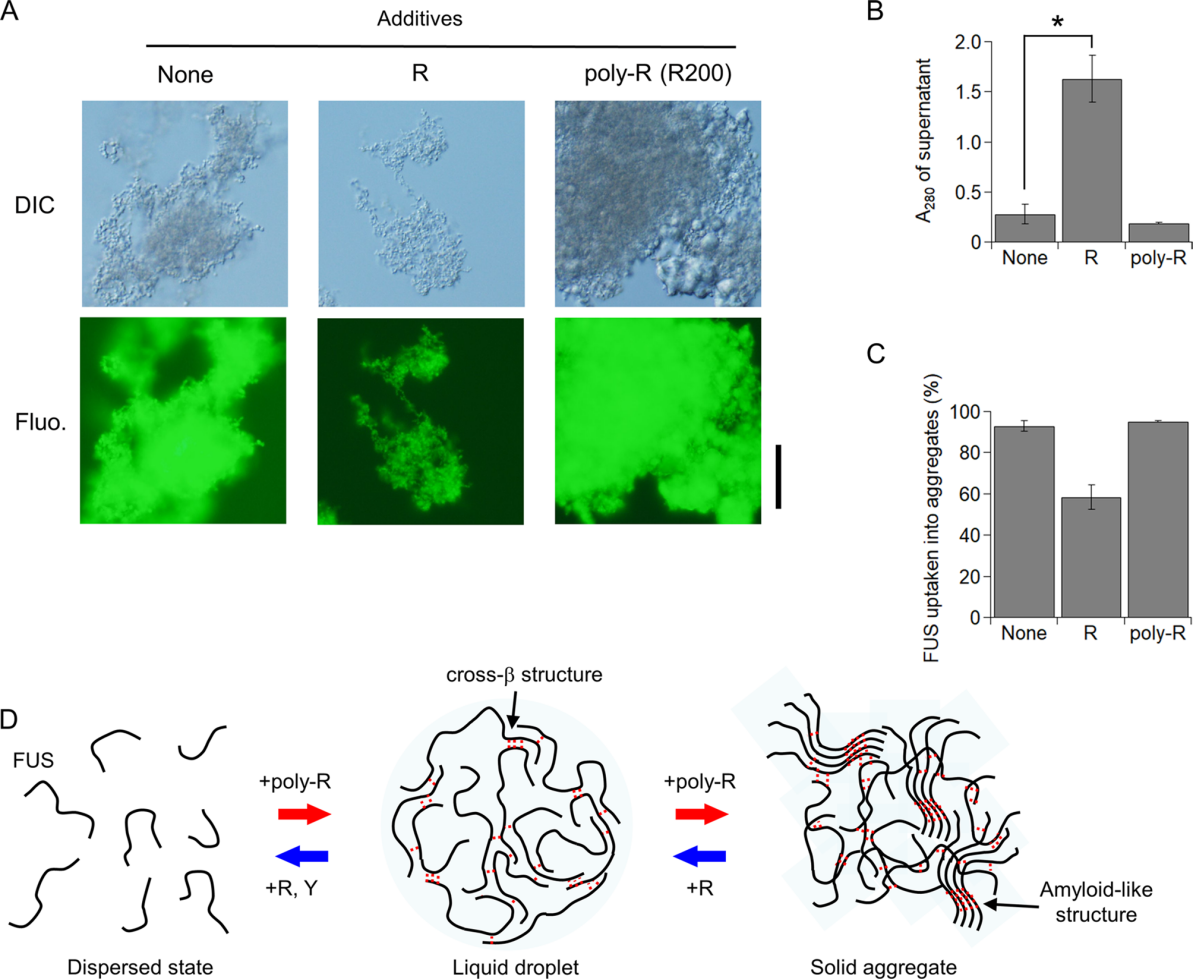
Arg additive suppressed the aggregation formation of FUS, while poly-Arg promoted it. (**A**) Effect of Arg and poly-Arg additives to FUS aggregate formation detected by DIC and fluorescence microscopy. The aggregates were formed after MBP-tag cleavage in the absence of dextran. The fluorescence of amyloid sensitive PicoGreen was detected. "None" denotes the absence of additives. Scale bar denotes 20 µm. (**B**) Absorbance of soluble fraction at 280 nm in FUS aggregate solution in the absence/presence of Arg and poly-Arg additives. Asterisk indicates a significant difference (*p* = 0.01, two-tailed t-test). (**C**) Percentage of FUS molecules taken up into aggregates in the absence/presence of Arg and poly-Arg additives. In (**B**, **C**), error bars represent the standard errors (*N* = 3). (**D**) Schematic diagram of effect of additives on the liquid droplets and solid aggregates. Dashed red lines represent intermolecular interactions.

Arg and poly-Arg might affect the formation of solid aggregates of FUS from the liquid droplets, because Arg and poly-Arg affected the droplet formation significantly. To form the solid aggregate of FUS, we evaluated several in vitro conditions by changing the time and temperature of incubation and/or cleavage of the MBP tag and selected the following condition: the droplet formation of FUS was formed by incubating MBP-FUS at 30 µM in the presence of TEV protease at 30 °C for 20 min, and solid aggregates of FUS were induced by incubating the sample at 70 °C for 2 h. The DIC image of FUS in the absence of additives showed non-spherical solid aggregates (Fig. 4A). The amyloid fluorescence probe, PicoGreen^25^, exhibited a high fluorescence intensity for the aggregates, implying amyloid formation in the FUS aggregates (Fig. 4A). The electron microscopy showed network structures composed of fibrous structures in non-spherical solid aggregates (Supplementary Fig. S8). In the presence of the Arg additive, the size of the FUS aggregates decreased (Fig. 4A). Thin filament structures in the aggregates were observed. In contrast, the poly-Arg (R200) additive increased the aggregate size (Fig. 4A). Similar result was obtained after incubation of MBP-FUS without MBP-tag cleavage for a week at 4 °C (Supplementary Fig. S9). In both cases, the fluorescence images suggested the presence of amyloids in the aggregates (Fig. 4A).

To determine the percentage of FUS molecules incorporated into the aggregate, we determined the concentration of soluble molecules in the supernatant by measuring the OD at 280 nm after centrifuging the samples (Fig. 4B). The OD at 280 nm (absorbance by FUS plus scattering) was corrected by subtracting the OD at 350 nm (scattering). In the absence of additives, 7% of the FUS molecules (corresponding to 2.1 µM) were in solution, indicating that 93% of the FUS molecules was included into the aggregate (Fig. 4C). When poly-Arg was added, the percentage of FUS in the aggregate slightly increased to 95%. In contrast, the Arg additive significantly reduced the percentage in the aggregate to 58%. Overall, the results demonstrated that the Arg additive suppresses the FUS aggregate formation, while poly-Arg promotes it. The similarity in the effect of the additives for the droplets and aggregates implies that aggregate regulation is achieved through regulation of the intermediate state (liquid droplets) (Fig. 4D).

### QQQQ and NNNN additives at high concentrations drove liquid droplets of FUS to solid aggregations

**Figure 5:**
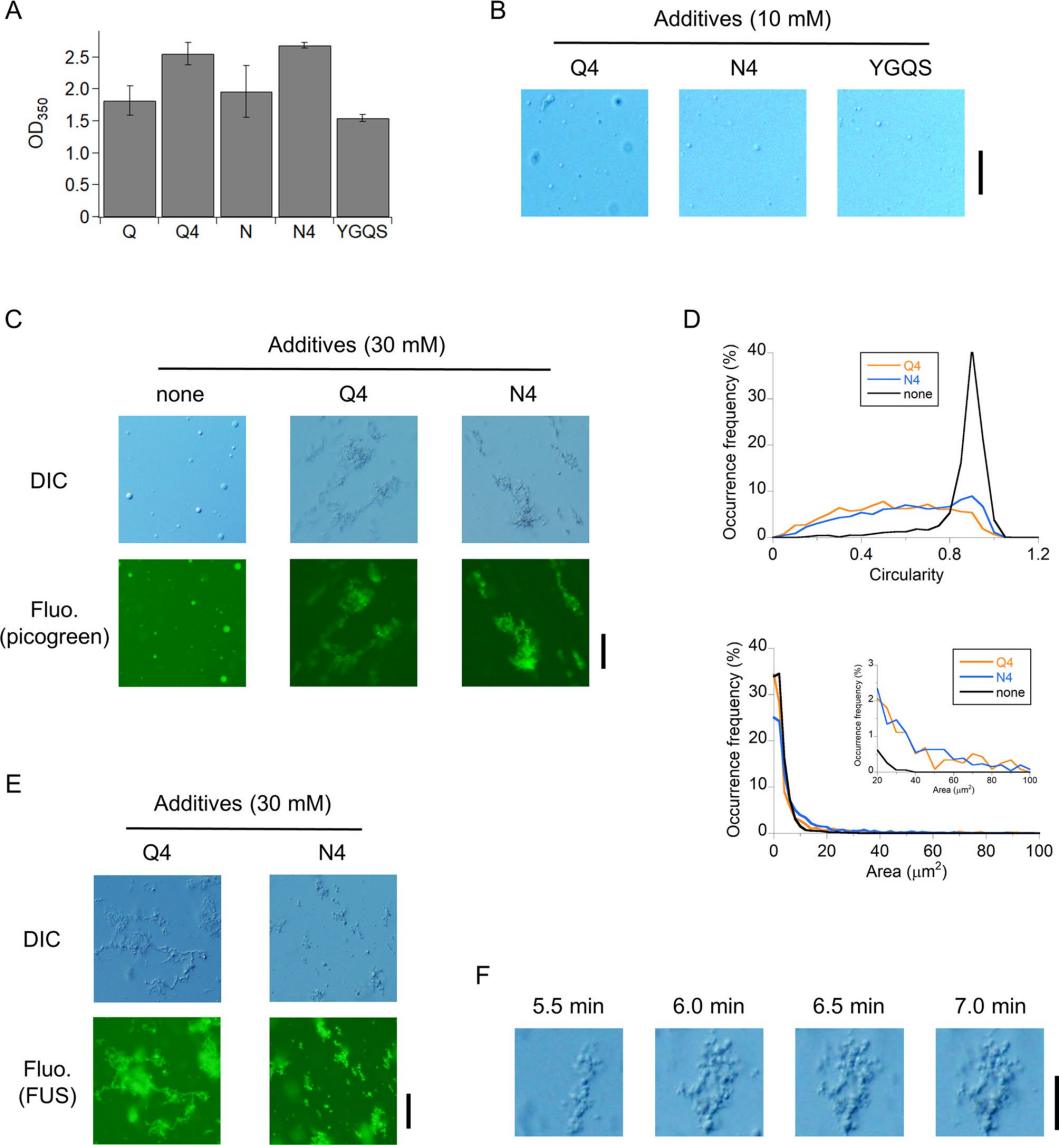
QQQQ and NNNN additives converted droplets of FUS into aggregates. (**A**) Effect of 4-mer peptides on droplets of MBP-FUS detected as the scattering at 350 nm (low concentrations). The solution contained 5 µM MBP-FUS plus 40 mM Gln or Asn or 10 mM 4-mer peptides in 140 mM KCl and 80 mg/mL dextran. Error bars represent the standard errors (*N* = 3). (**B**) DIC images of MBP-FUS solutions in the presence of 4-mer peptides at low concentrations. The solution conditions were the same as in (**A**). Scale bar denotes 10 µm. (**C**) DIC and fluorescence images of MBP-FUS solution in the presence/absence of Q4 or N4 at high concentrations. The solution contained 15 µM MBP-FUS plus 30 mM Q4 or N4 in 183 mM KCl and 65 mg/mL dextran. Scale bar denotes 10 µm. The fluorescence of amyloid-sensitive PicoGreen was detected. None denotes the absence of additives. Scale bar denotes 20 µm. (**D**) Distributions of cross-section area and circularity of individual droplets or aggregates of MBP-FUS in the DIC images in the presence of Q4 (*N* = 1170) or N4 (*N* = 2528) at high concentrations or in the absence of the additives (*N* = 3419). The solution conditions were the same as in (**C**). (**E**) Uptake of MBP-FUS into the aggregates evaluated using DIC and fluorescence microscopy; 0.1 µM MBP-FUS-Alexa488 was added to a solution of non-labeled MBP-FUS, as described in (**C**). Scale bar denotes 20 µm. (**F**) Time-lapse DIC images for aggregate formation of MBP-FUS in the presence of N4 at high concentrations. The time after mixing MBP-FUS and N4 is displayed. The solution conditions were the same as in (**C**). Scale bar denotes 10 µm.

Since the cross-beta structures formed by intermolecular H-bonds participate in the formation of liquid droplets, as well as gel-like droplets and amyloid fibrils^7,10^, we evaluated the effect of QQQQ (Q4) and NNNN (N4), amyloid-related peptides, on the droplet formation of MBP-FUS at 5 µM. Q4 and N4 at 10 mM, corresponding to 40 mM of monomer, increased the scattering intensity to some extent, suggesting the enhancement of droplet formation (Fig. 5A), where the OD_350_ of Q4 or N4 was subtracted from that of the sample containing MBP-FUS plus Q4 or N4, because Q4 and N4 produced a small scattering effect. In contrast, YGQS, composed of four residues frequently observed in LLPS proteins, did not affect droplet formation. The DIC images demonstrated spherical droplets in the presence of the three different peptides (Fig. 5B). The cross-section area and circularity analysis of the MBP-FUS droplets indicated no significant effect upon the addition of Q4 and N4 (Supplementary Fig. S10). Q4 and N4 additives at low concentrations slightly enhanced FUS droplet formation without modulating the shape and size distribution.

We examined the effect of Q4 and N4 additives in MBP-FUS droplets at higher concentrations. The solution contained 15 µM MBP-FUS and 30 mM Q4 or N4. Non-spherical aggregates of MBP-FUS were generated in the presence of Q4 and N4 additives (Fig. 5C). The aggregates exhibited a high fluorescence intensity from PicoGreen, which suggested amyloid formation in the aggregates (Fig. 5C). As a control, spherical droplets were detected in the absence of Q4 or N4 additives (Fig. 5C). Additionally, the droplets exhibited high fluorescence intensity from PicoGreen, implying amyloid formation in the FUS droplets, consistent with the results of a previous study^26^. The circularity distribution of each droplet or aggregate became broader in the presence of Q4 or N4 additives, supporting non-spherical aggregates induced by the additives (Fig. 5D). In addition, the additives increased the frequency of droplets or aggregates with a cross-section area greater than 10 µm^2^ (Fig. 5D). Additionally, MBP-FUS labeled with Alexa488 was incorporated into the aggregates, confirming the aggregation of MBP-FUS (Fig. 5E). Thin filament structures in the aggregates supported amyloid formation, especially in the presence of Q4 (Fig. 5E). The time-lapse DIC images demonstrated that MBP-FUS formed non-spherical aggregates; subsequently, the aggregates assembled and became larger (Fig. 5E). Long storage with Q4 or N4 solution promoted the aggregation of MBP-FUS. In addition, Q4 and N4 themselves formed small non-spherical aggregates containing amyloids under this condition (Supplementary Fig. S11). Therefore, we propose that the small amyloid core formed by Q4 and N4 induced the conversion of the FUS droplets to FUS aggregates.

## Discussion

In this study, we propose an experiment-guided protocol for designing peptides that can regulate the formation of liquid droplets and solid aggregates. Owing to the tremendous number of peptide candidates, experimental screening may not be successful. In contrast, the developed protocol can efficiently explore droplet-regulating peptides. The protocol is essentially composed of two simple steps, and it can be widely applied to various proteins of interest. The first step is evaluation of the effect of 19 amino acid additives on the droplets of a protein of interest and identification of effective amino acid that can suppress droplets, such as Arg or Tyr for FUS. The second step is synthesis of the polymer of the identified amino acid. The synthesized polymer would bridge at least two protein molecules and promote droplet formation, such as poly-Arg for FUS. The use of the effective amino acids in designing the peptide can significantly reduce the pool of potential peptide candidates. Using this protocol, we succeeded in identifying additives that suppressed and enhanced the formation of FUS droplets and aggregates.

To alter the state of proteins from liquid droplets to aggregates or amyloids, we need to consider intermolecular β-sheet formation between the backbones. The above protocol, focusing on the side chain effect, does not consider this aspect. Nevertheless, the β-sheet-forming peptide serves for this purpose. The Q-rich region of amyloid-forming proteins, such as the LC domain of FUS forms intermolecular β-sheets, which leads to aggregation. The β-sheet-forming peptide supports this intermolecular β-sheet formation for the protein of interest. N4 or Q4 can form amyloids by itself^27–29^ (Supplementary Fig. S11) and induce FUS to form liquid droplets at low concentrations and to convert from the droplet state to the aggregate state, including amyloids, at high concentrations (Fig. 5). This phenomenon may be similar to cross-amyloid aggregation and cross-seeding: co-aggregation occurs between different proteins or peptides^30^. For example, poly-Glu enhances Tau aggregation^31^. We propose that the cross-β structure of FUS is induced near the small amyloid core of Q4 and N4 and that it propagates over other FUS molecules, thus promoting the aggregation of FUS.

The experiment-guided protocol and MD simulations provide information on the design grammar for LLPS-regulating peptides. As an additive, poly-Arg, in this study, accelerated FUS droplet formation more effectively than poly-Lys. Arg and Lys in the polymer possess a positive charge, exhibiting cation–π and electrostatic interactions with FUS. Arg may exhibit a stronger cation–π interaction than Lys^32^. In addition, Arg enables π–π interactions via its π-bonded guanidium group^33,34^. Such different interactions between Arg and Lys resulted in the Arg additives forming a greater number of contacts with Tyr and Phe residues of FUS compared to Lys additives, according to MD simulation (Fig. 1G). Consistent with this result, delocalization of positive charge within the guanidium group of Arg can result in hydrogen bond formation with the backbone of FUS^35^ (Fig. 1G).

Poly-Arg additive promotes the droplet formation of FUS by contacting to Tyr residues of the LC domain, Asp residues of the RGG1 domain, and Asp/Glu residues of the RRM domain. These interactions are similar with those formed in FUS droplets, such as Arg residues of the RNA-binding domain and Tyr residues of the LC domain^9,10^ and electrostatic interactions with Asp^9^. Accordingly, poly-Arg bridges the key residues of FUS which participate in the droplet formation.

In conclusion, we propose an experiment-guided protocol for characterizing the design grammar of peptides that can regulate the formation of liquid droplets and solid aggregates. The design grammar would depend on the proteins of interest; however, Arg and poly-Arg, which were identified as effective additives in this study, might serve for other proteins, because the LLPS proteins provide many donors for cation–π, π–π, and electrostatic interactions with these additives. In drug design, this protocol could be used for the primary design of peptides targeting disease-related proteins formed via liquid droplets. The designed peptide might be further optimized toward achieving aggregate suppression with high affinity using other established methods such as phage display.

## Materials and methods

### FUS samples

We prepared the MBP-FUS sample composed of 6 × His-tag, MBP tag, TEV cleavage sequence, and human FUS wild type*,* 6 × His-MBP-TEV-FUS, following the protocol reported in an earlier study^12^ with some modifications. The pTHMT plasmid containing 6 × His-MBP-TEV-FUS was purchased from Addgene (no. 98651). For labeling with a fluorescent dye, we prepared a gene containing the cys mutant of FUS, in which cysteine was introduced at the C-terminus using a PrimeSTAR Mutagenesis Basal Kit (TaKaRa), because no cysteine residue exists in wild-type FUS. *Escherichia coli* cells with the plasmid were cultured in LB media with 50 µg/mL Kanamycin at 37 °C. When the OD_600_ reached 0.8, we added 0.5 mM IPTG and cultured at 18 °C for ~ 12 h. The collected cells were lysed by ultra-sonication in a solution containing 50 mM HEPES, 1.5 M NaCl, 10% glycerol, and 2 mM DTT (pH 7.4). A protease inhibitor cocktail was added to prevent the degradation of samples (cOmplete, Mini, Sigma-Aldrich). To remove DNA and RNA from the MBP-FUS sample, 1.5 M NaCl was used. The MBP-FUS samples were purified using HisTrap (HisTrap FF crude; GE Healthcare) and heparin columns (HiTrap Heparin HP; GE Healthcare) without cleavage of the MBP tag. The purity was confirmed using SDS-PAGE.

### Amino acids and peptides

Amino acids were purchased from FUJIFILM Wako Pure Chemical Corporation and Nacalai Tesque. Poly-L-Arg and poly-L-Lys were obtained from Sigma-Aldrich and used without further purification. R2, R3, and R10 were purchased from GenScript. R10, R5, Q4, N4, and YGQS were synthesized using a standard Fmoc-based solid-phase peptide synthesis. These peptides were cleaved from the resin using trifluoroacetic acid at 95%, water at 2.5%, and triisopropylsilane at 2.5%. The cleaved peptides were precipitated in cold diethyl ether. The resultant peptides except for R10 were dissolved in 0.1% TFA (HCl for R10) and lyophilized. The purity and identity of each peptide were verified using HPLC and mass spectrometry (LTQ Orbitrap XL ETD, Thermo Fisher Scientific Inc.).

### Labeling with a fluorophore

The cysteine mutant of MBP-FUS was labeled with Alexa488 (Thermo Fisher) in a solution containing 100 mM Tris, 500 mM KCl, and 1 mM TCEP using maleimide chemistry and purified using gel filtration (PD MiniTrap G-25; GE Healthcare). Because MBP-FUS, bound nonspecifically to unreacted dyes, was eluted, the bound dyes were further removed using a centrifugal filter (Amicon Ultra-4, Millipore) by increasing the concentration of KCl to 1.5 M. The N-terminus of poly-R was labeled with Alexa488 in a solution containing 100 mM NaHCO_3_ and 500 mM NaCl (pH 8.3) using succinimidyl ester chemistry and purified using gel filtration. The labeling ratios were determined to be 1.1 for MBP-FUS-Alexa488, according the absorbance values at 280 nm and 495 nm, and 0.96 for poly-R-Alexa488, according to the absorbance values at 220 nm and 495 nm.

### Sample preparation for FUS droplets

To examine the dextran dependence of FUS droplets, we used solutions containing 5 µM MBP-FUS, 100 mM Tris, 140 mM KCl, and 1 mM DTT at pH 7.4. We used dextran with a molecular weight between 450,000 and 650,000 (Sigma-Aldrich). To evaluate the effect of amino acids and peptides on FUS droplets, we used solutions containing 5 µM MBP-FUS, 100 mM Tris, 140 mM KCl, 1 mM DTT, and 90 mg/mL Dextran at pH 7.4. To analyze the effect of Q4 and N4 at high concentrations, we used solutions containing 15 µM MBP-FUS, 30 mM Q4 or N4, 100 mM Tris, 183 mM KCl, 1 mM DTT, and 65 mg/mL Dextran at pH 7.4. To evaluate the uptake of poly-R into FUS droplets, we used solutions containing 5 µM MBP-FUS, 0.1 µM poly-R-Alexa488, 100 mM Tris, 140 mM KCl, 1 mM DTT, and 80 mg/mL dextran at pH 7.4. These solutions were prepared through a fivefold dilution of a stock solution containing 500 mM KCl at pH 7.4 and incubated for 10 min at 20 °C before the analyses. For the regulation of FUS droplets by additives in the absence of the MBP tag, the droplet formation of FUS was initiated through the addition of TEV protease (TurboTEV Protease; Accelagen) at 769 µg/mL in a solution containing 15 µM MBP-FUS, 100 mM Tris, and 140 mM KCl (pH 7.4). After incubation for 20 min at 20 °C, 40 mM Arg and 8.3 mg/mL poly-R were added as additives; the final concentration of FUS was 5 µM. The solutions were incubated for 5 min at 20 °C, and the ODs were measured at 350 nm.

### Aggregate preparation of FUS

Droplets of FUS were formed by incubating the solution containing 30 µM MBP-FUS, 100 mM Tris (pH 7.4), 319 mM KCl, and TEV protease at 60 µg/mL at 30 °C for 20 min. Subsequently, solid aggregates of FUS were formed by incubating the sample at 70 °C for 2 h. Prior to the fluorescence measurements, PicoGreen (Invitrogen) was added by 300-fold dilution of the purchased stock.

### Scattering measurements

We analyzed light scattering from the droplets of MBP-FUS or FUS by measuring the OD_350_ using an absorbance spectrometer (NanoDrop One; Thermo Fisher).

### DIC microscopy

We used the DIC mode of the inverted microscope (IX-73; Olympus, Tokyo, Japan), as described previously^36^. The sample solution was casted on the coverslip and covered by the slide glass. The DIC images were captured at 40 × or 60 × magnification at 21 °C. The cross-section area and circularity of the droplets were calculated using the Image J software.

### Fluorescence microscopy for in vitro measurements

To evaluate the uptake of poly-R-Alexa488 or MBP-FUS-Alexa488 into droplets and aggregates of non-labeled MBP-FUS, we used the fluorescence mode of the inverted microscope (IX-73; Olympus)^36^. The excitation and emission wavelengths were 470–490 nm and 515–550 nm, respectively. Fluorescence images were captured at 21 °C.

### MD simulation

MD simulations were conducted to investigate interactions between MBP-FUS and several amino acid additives. Because the MBP-FUS structure was not solved, it was generated from the crystal structures of MBP (PDB ID: 1Y4C), the RRM domain (PDB ID: 2LA6), and the ZnF domain (PDB ID: 6G99)^37^ of FUS. The disordered regions of FUS were modeled as extended structures. Through connecting these structured and disordered domains, the overall structure of MBP-FUS was constructed. To equilibrate the structure, 300 ns MD simulation was performed with implicit solvent. The protein was described using the AMBER14SB force field^38^ and Generalized Born energy^39^.

Next, the protein was simulated in a water/amino acid additive box. These molecules were placed in a dodecahedron box with 24.1 nm sides. The system contained one molecule of MBP-FUS, 311,439 water molecules, 589 amino-acid additive molecules corresponding to 100 mM, and 895 sodium and chloride ion molecules corresponding to 150 mM. When the total charge of the system was not neutral, additional sodium or chloride ions were also included. The protein and amino acid additives were described using the AMBER99SB force field^40^. Since the zwitterion form of amino acids is not prepared in this force field, we built their molecular model with restrained electrostatic potential (RESP) charges^41^ (Supplementary text). Water was described by the TIP3P model^42^. Sodium and chloride ion models were obtained from the literature^43^.

After energy minimization, constant-pressure and constant-temperature (NPT) MD simulations were performed at 1 bar and 300 K for 0.1 ns for equilibrating the system, and the production runs were performed for 100 ns. Three runs were performed for each amino acid solution with different initial velocities. The Parrinello-Rahman method was used to maintain pressure during the NPT simulation^44^. The Langevin dynamics was used to maintain the temperature with water viscosity set to 2 ps^−1^. The covalent bonds of hydrogen atoms in proteins were constrained using the LINCS method^45^, and the integration time step was 2.0 fs. MD simulations were performed using GROMACS 2018.1^46^.

For the contact evaluation, 10–100 ns trajectories were used. A contact was defined as a distance of less than 6.5 Å between the centers of two side chains, except for Gly, for which Cα was used.

#### Cell measurements

Primary cultures of mesencephalic neurons were prepared as previously described^47,48^. GFP-tagged human FUS (#HG16569-ACG) was purchased from Sino Biological Inc. The expression vector was transfected into cultured dopaminergic neurons using Lipofectamine 3000 (Thermo Fisher Scientific). 48 h after the transfection, cells were exposed to 100 µM H_2_O_2_ with or without R10 (300 µM) or arginine (300 µM) for 6 h, and then fixed by 4% paraformaldehyde for 30 min. For immunocytochemistry, the cells were further incubated with 0.1% Triton X-100 for 15 min. After pre-blocking with 5% goat serum in phosphate-buffered saline (PBS) for 1 h, they were incubated overnight at 4 °C with the following primary antibodies: rabbit anti-TH affinity-purified polyclonal antibody (1:400; Millipore). After washing with PBS, the cells were incubated with either Alexa Fluor 546-conjugated secondary antibodies (1:500 dilution, Invitrogen). Images were acquired at 37 °C using a confocal laser scanning microscope (TCS SP8, Leica Microsystems). The areas of FUS clusters, defined by an area above 1 μm^2^ in TH^+^ cells, were calculated using Image J software.

## Supplementary Information

Supplementary Information
