# Supplementary material for: Characterization of design grammar of peptides for regulating liquid droplets and aggregates of FUS
Source: Sci Rep. 2021 Mar 23;11:6643. doi: 10.1038/s41598-021-86098-1 (PMC7988016; doi:10.1038/s41598-021-86098-1)
Supplement: Supplementary file 1 — Supplementary Information [file 41598_2021_86098_MOESM1_ESM.docx]

Supplementary Information for

**Characterization of design grammar of peptides for regulating liquid droplets and aggregates of FUS**

Kiyoto Kamagata^1,2,3*^, Rika Chiba^1,2^, Ichiro Kawahata^4^, Nanako Iwaki^1,3^, Saori Kanbayashi^1^, Kana Maeda^1,3^, Hiroto Takahashi^1^, Atsushi Hirano^5^, Koji Fukunaga^4^, Keisuke Ikeda^6^, and Tomoshi Kameda^7^

^1^Institute of Multidisciplinary Research for Advanced Materials, Tohoku University, Katahira 2-1-1, Aoba-ku, Sendai 980-8577, Japan

^2^Graduate School of Life Sciences, Tohoku University, Sendai 980-8578, Japan

^3^Department of Chemistry, Graduate School of Science, Tohoku University, Sendai 980-8578, Japan

^4^Department of Pharmacology, Graduate School of Pharmaceutical Sciences, Tohoku University, Sendai 980-8578, Japan

^5^Nanomaterials Research Institute, National Institute of Advanced Industrial Science and Technology (AIST), Tsukuba, Ibaraki 305-8565, Japan.

^6^Department of Biointerface Chemistry, Faculty of Pharmaceutical Sciences, University of Toyama, 2630 Sugitani, Toyama 930-0194, Japan.

^7^Artificial Intelligence Research Center, National Institute of Advanced Industrial Science and Technology (AIST), Koto, Tokyo 135-0064, Japan

The first two authors should be regarded as joint First Authors.

*Corresponding author: Kiyoto Kamagata

Institute of Multidisciplinary Research for Advanced Materials, Tohoku University, Katahira 2-1-1, Aoba-ku, Sendai 980-8577, Japan

TEL: +81-22-217-5843/FAX: +81-22-217-5842

e-mail: kiyoto.kamagata.e8@tohoku.ac.jp


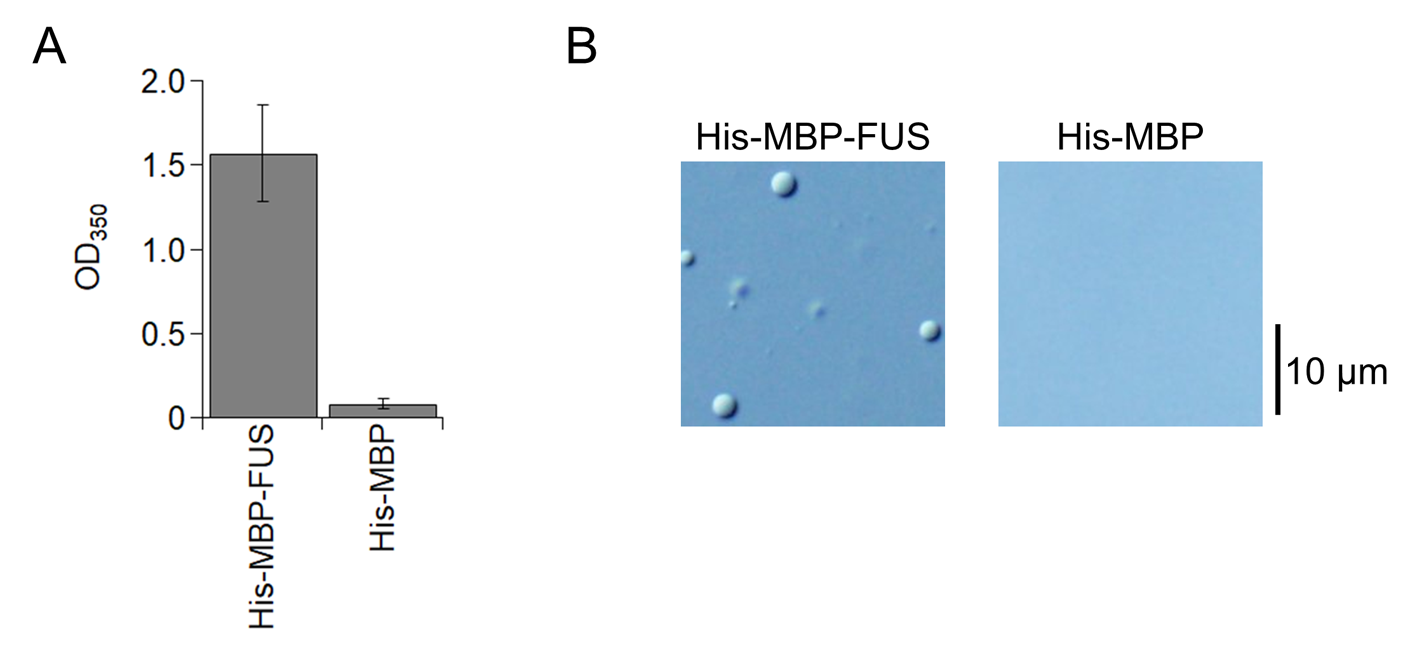


**Fig. S1.** Scattering measurement (A) and DIC microscopy (B) of His-MBP-FUS and His-MBP solution. His denotes His tag. The solutions included 100 mM Tris-HCl (pH 7.4), 140 mM KCl, 1 mM DTT, 80 mg/mL Dextran, and 5 μM His-MBP or His-MBP-FUS. In panel (A), OD was detected as the scattering at 350 nm. The error bars denote the standard errors (*N* = 3).

**
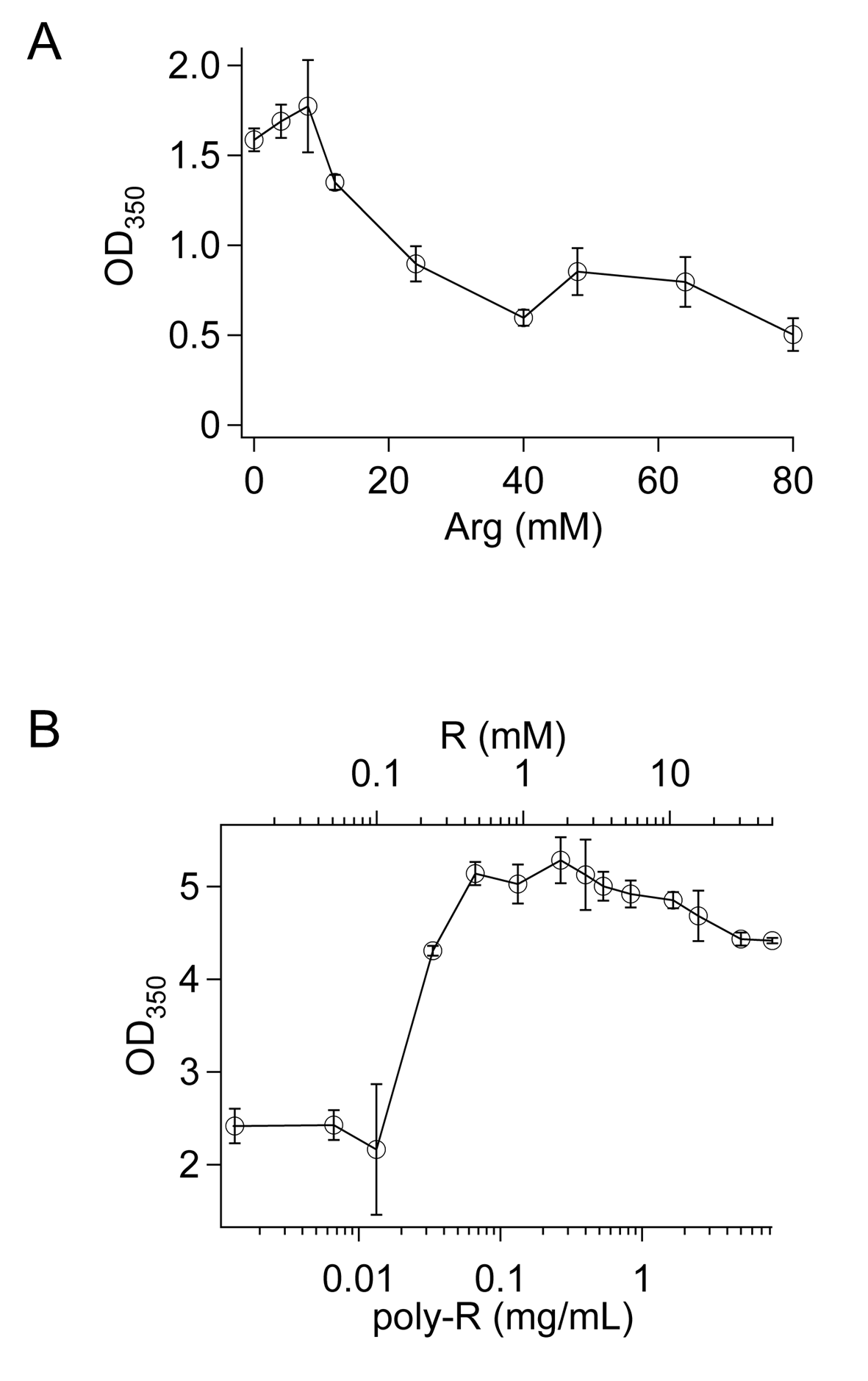
**

**Fig. S2.** (A) Scattering from MBP-FUS droplets at different concentrations of Arg additive. (B) Scattering from MBP-FUS droplets at different concentrations of poly-Arg additive. R concentration on top axis represents the monomer unit concentration. The solution contained 80 mg/mL Dextran. The error bars denote the standard errors (*N* = 3).

**
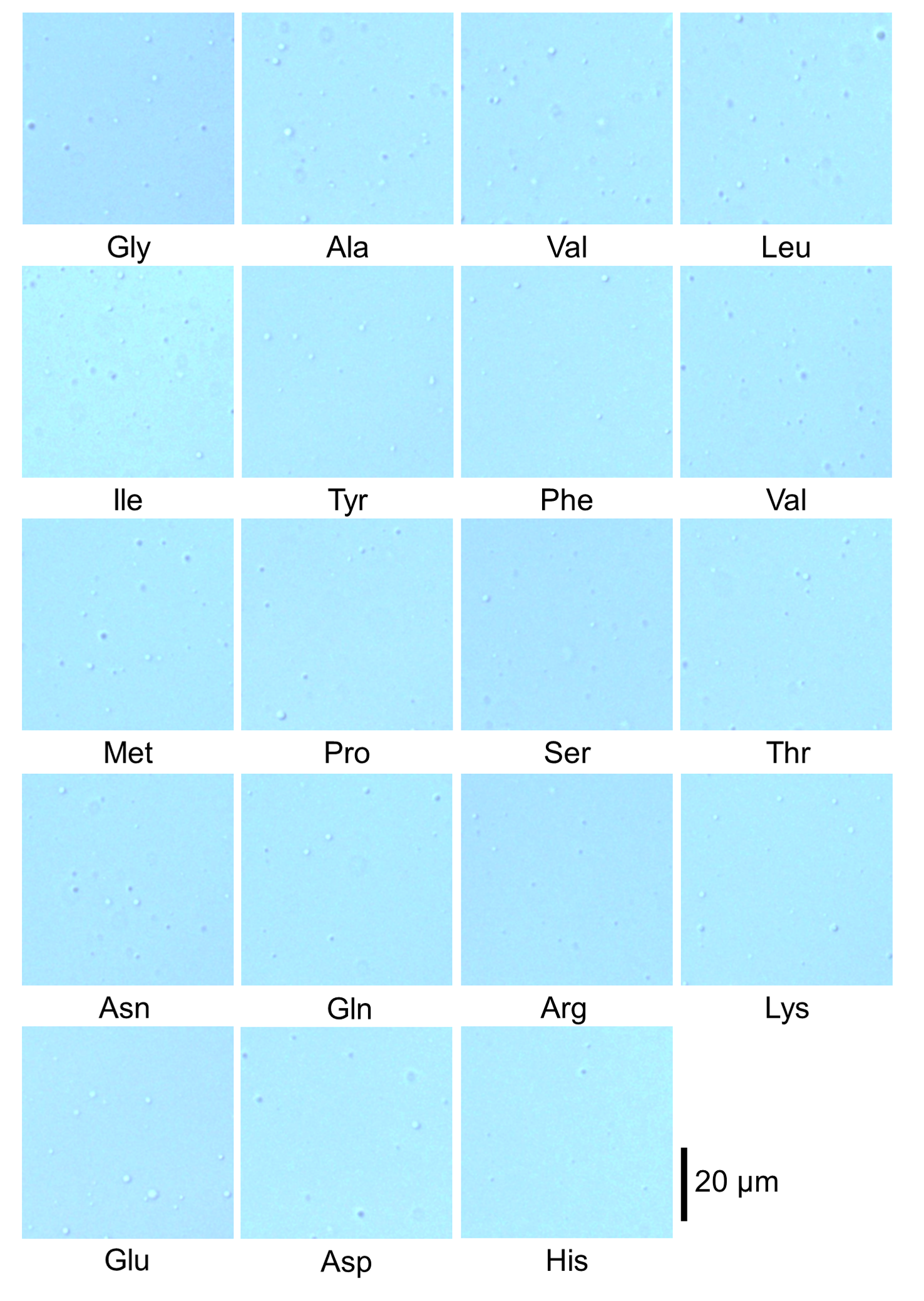
**

**Fig. S3.** Effect of 19 amino acids as additives for MBP-FUS droplet formation detected by DIC microscopy. The solution contained 80 mg/mL Dextran and 40 mM amino acids except for Tyr or 2 mM Tyr due to low solubility.


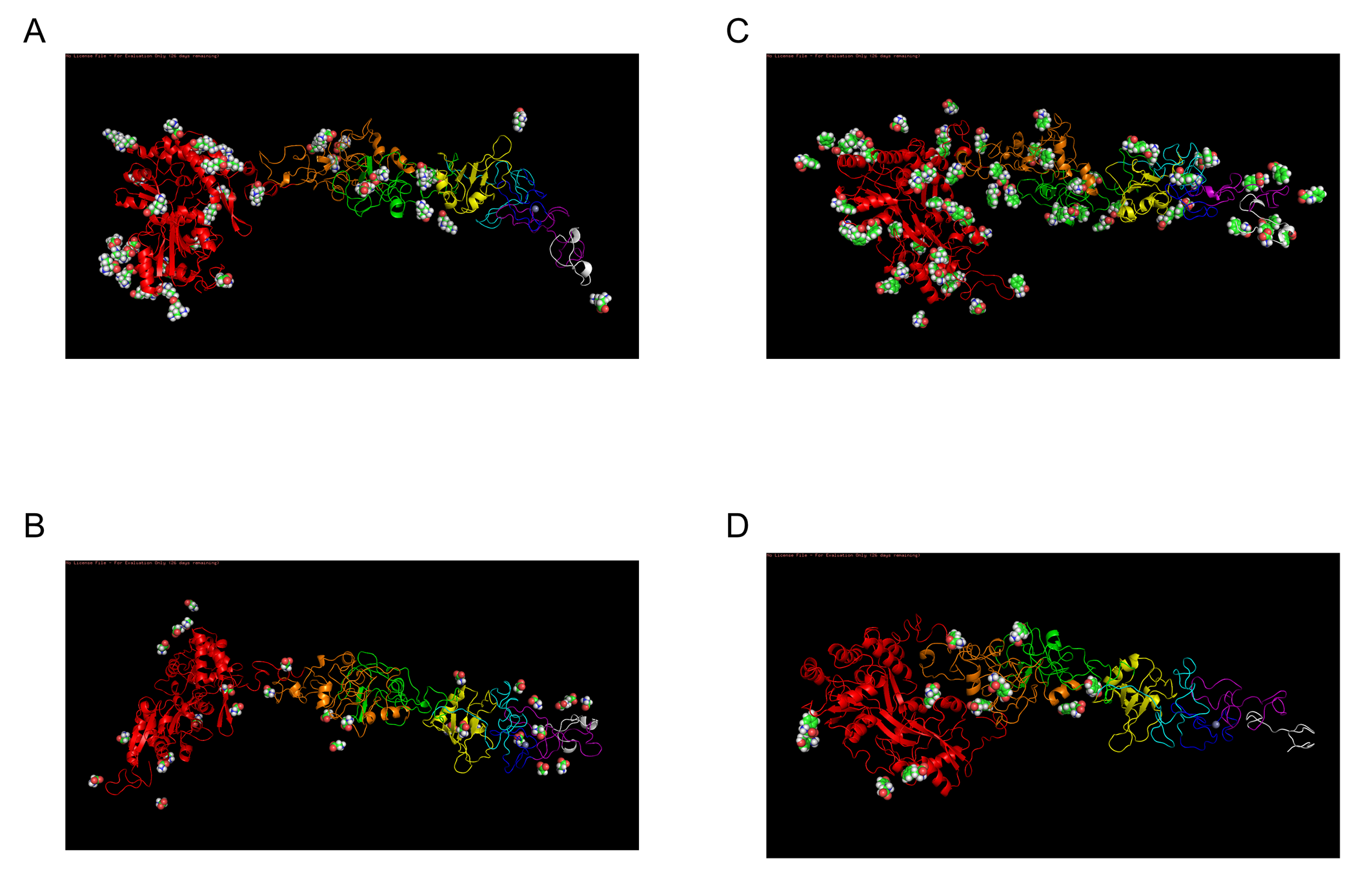


**Fig. S4.** Snapshots of MBP-FUS in the presence of Lys (A), Gly (B), Phe (C), and Tyr (D) additives in MD simulations. Snapshots at 100 ns are displayed. Red, orange, green, yellow, light blue, blue, purple and white ribbon structures denote MBP, LC, RGG1, RRM, RGG2, ZnF, RGG3, and PY-NLS domains of FUS, respectively. Space-filled structures represent amino acid additives contacting to MBP-FUS.


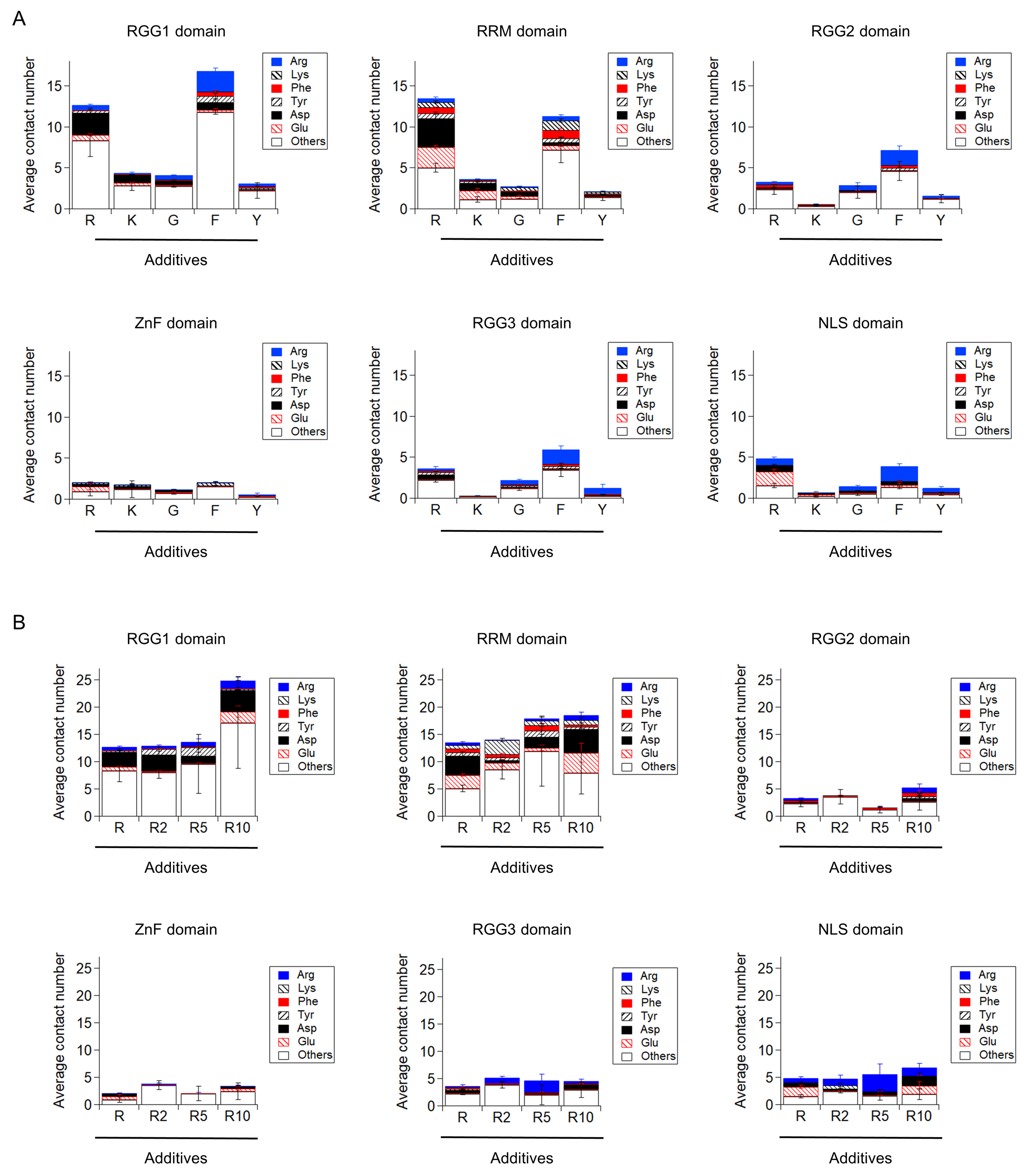


**Fig. S5.** Average number of contacts between the amino acid (A) or poly-Arg (B) additives and intact residues of RGG1, RRM, RGG2, ZnF, RGG3, and PY-NLS domains of FUS in the molecular dynamics simulations. The residues of FUS are classified into droplet-related residues plus Glu (colors or shaded) or others (blank).

**
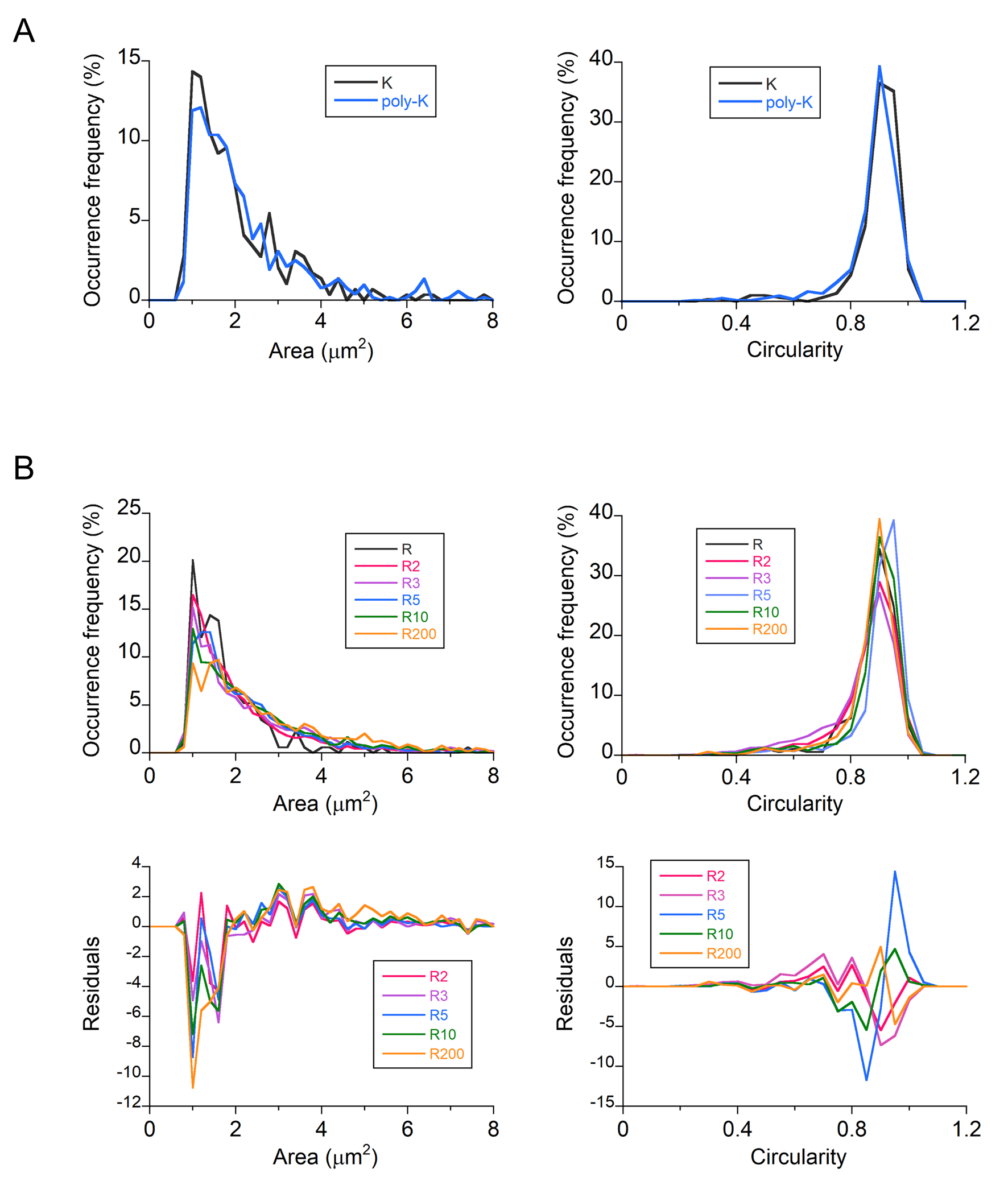
**

**Fig. S6.** Distributions of cross-section area and circularity of individual droplets of MBP-FUS in the presence of K and poly-K. K concentrations in monomer were set to 40 mM for monomers and polymers. 293 and 529 droplets in DIC images were analyzed for K and poly-K, respectively.


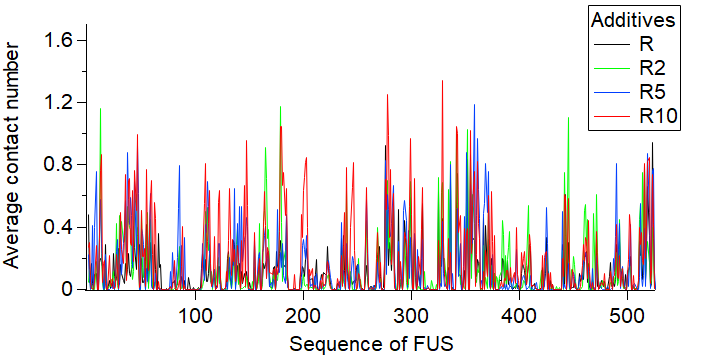


**Fig. S7.** Average contact number of Arg (R), R2, R5, and R10 additives to FUS sequence in MD simulations.


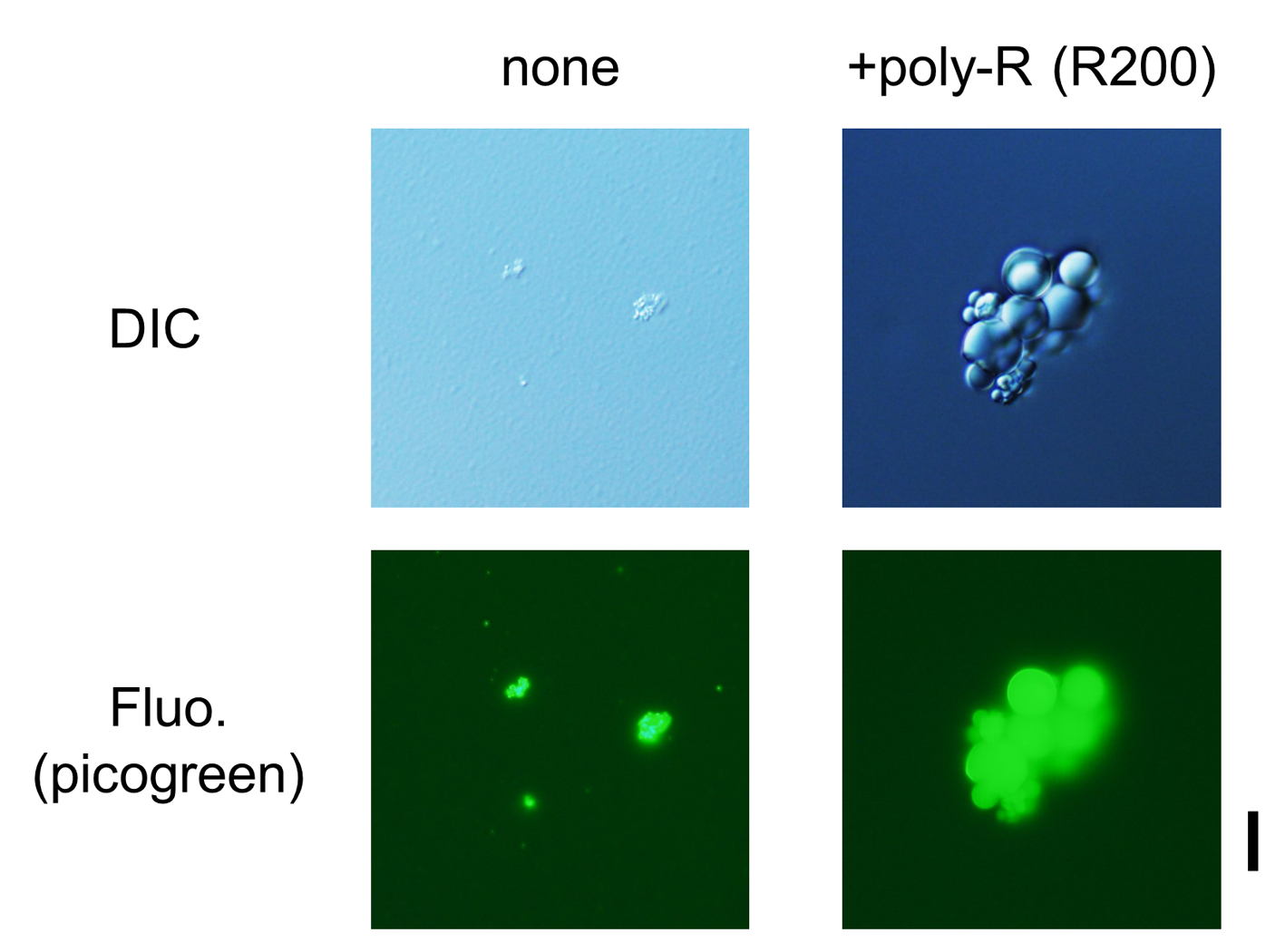


**Fig. S8.** DIC and fluorescence microscopic images of MBP-FUS in the presence and absence of poly-R after long-tern incubation. The solution contained 30 μM FUS, 100 mM Tris-HCl, 320 mM KCl, and 1 mM DTT (pH 7.4). poly-R was added to be 4.16 mg/mL. The data were taken after incubation for 1 week at 4°C. The fluorescence of amyloid sensitive Picogreen was detected. Scale bar denotes 20 µm.


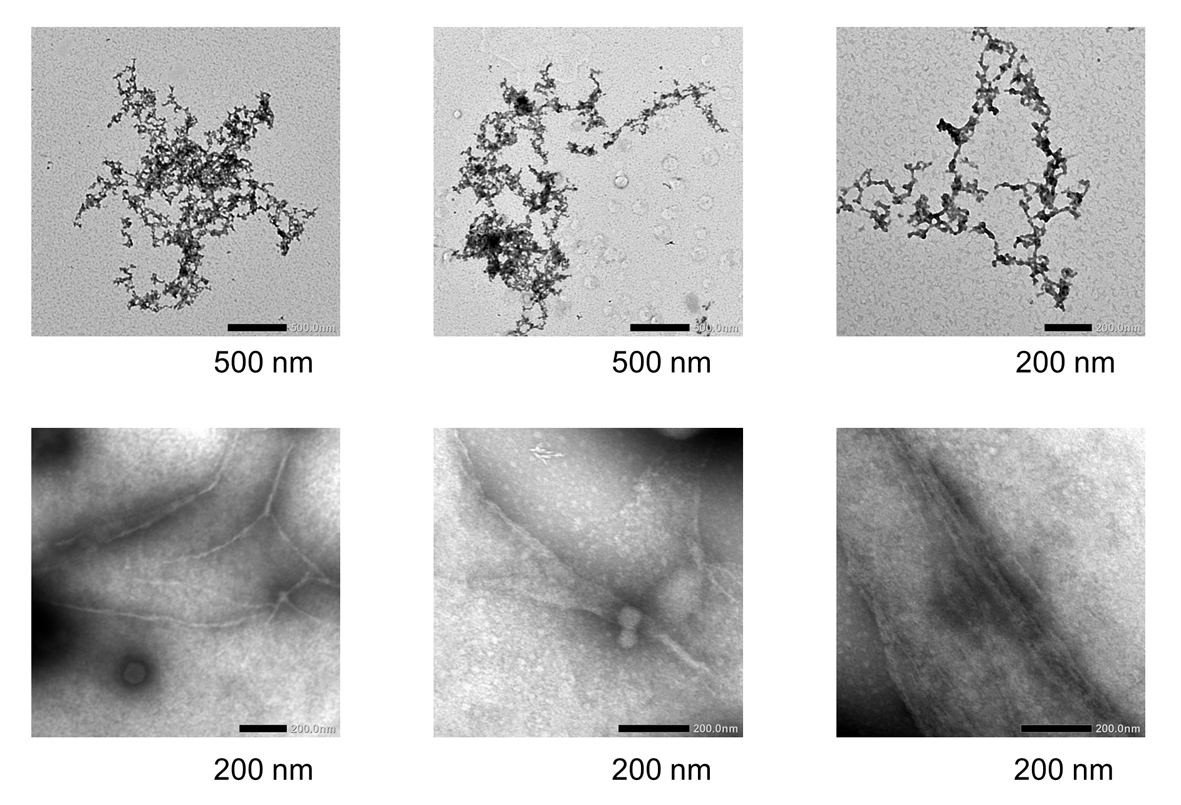


**Fig. S9.** Transmission electron microscopic images of non-spherical aggregate of FUS. Top images represent global network structures composed of fibrous structures with several~10 nm of width. Bottom images, obtained in small amounts, represent fibers or spherical clusters. The aggregate was placed on a Cu grid coated with a thin carbon ﬁlm (300 meshes) for 2 min. The excess solution on the grid was removed using a ﬁlter paper. The grid was negatively stained with 2% phosphotungstic acid solution (pH 6.5) for 30 s and dried after the removal of the excess solution. TEM images were obtained using a JEM-1400 transmission electron microscope (JEOL Inc., Tokyo, Japan) operated at 80 keV. The magnification was 10,000~30,000.


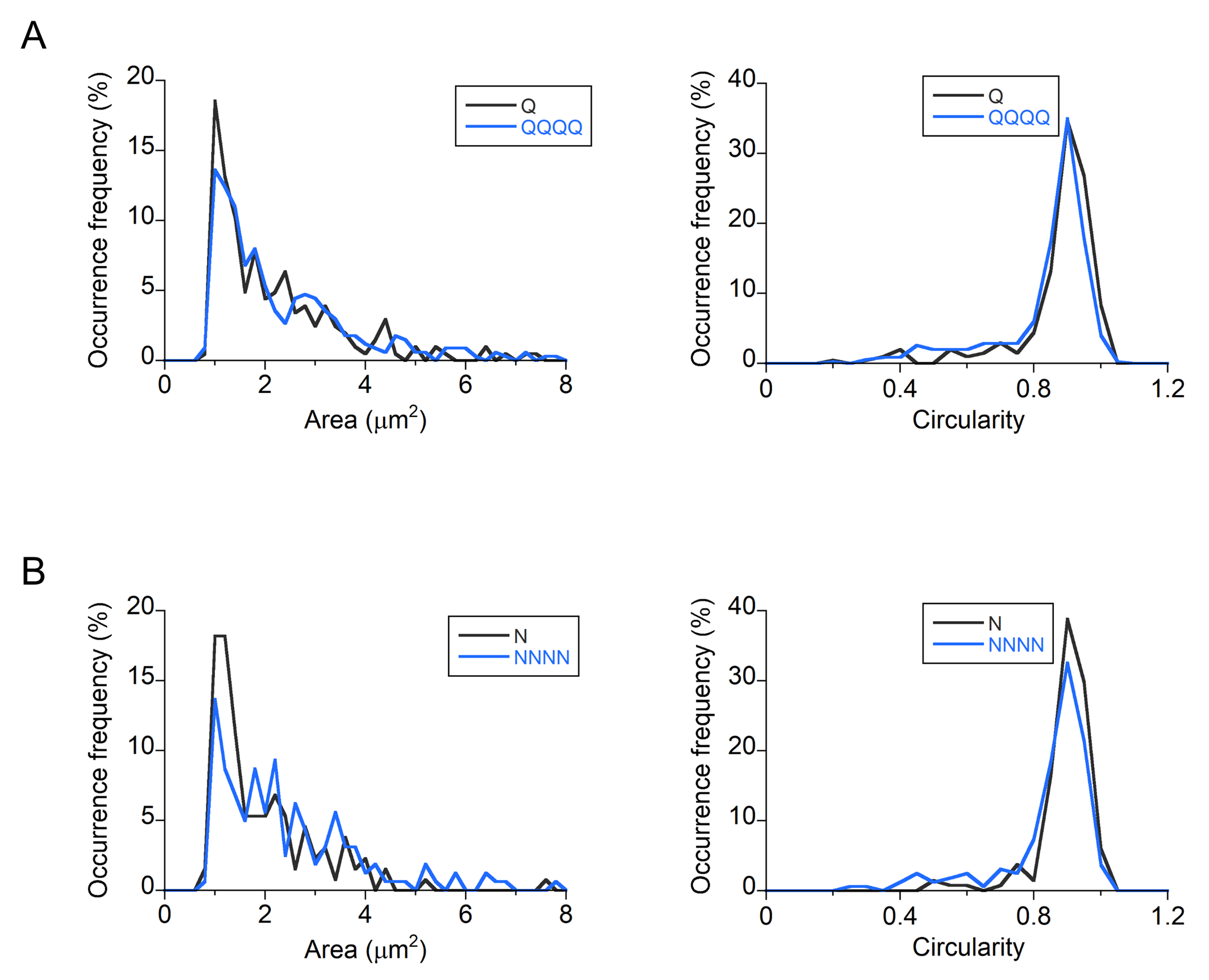


**Fig. S10.** Distributions of cross-section area (A) and circularity (B) of individual droplets of MBP-FUS in the presence of Q, Q4, N, and N4 at low concentrations. The solution contained 40 mM N or Q or 10 mM 4-mer peptides. 205, 352, 134, and 163 droplets in DIC images were analyzed for Q, Q4, N, and N4, respectively.


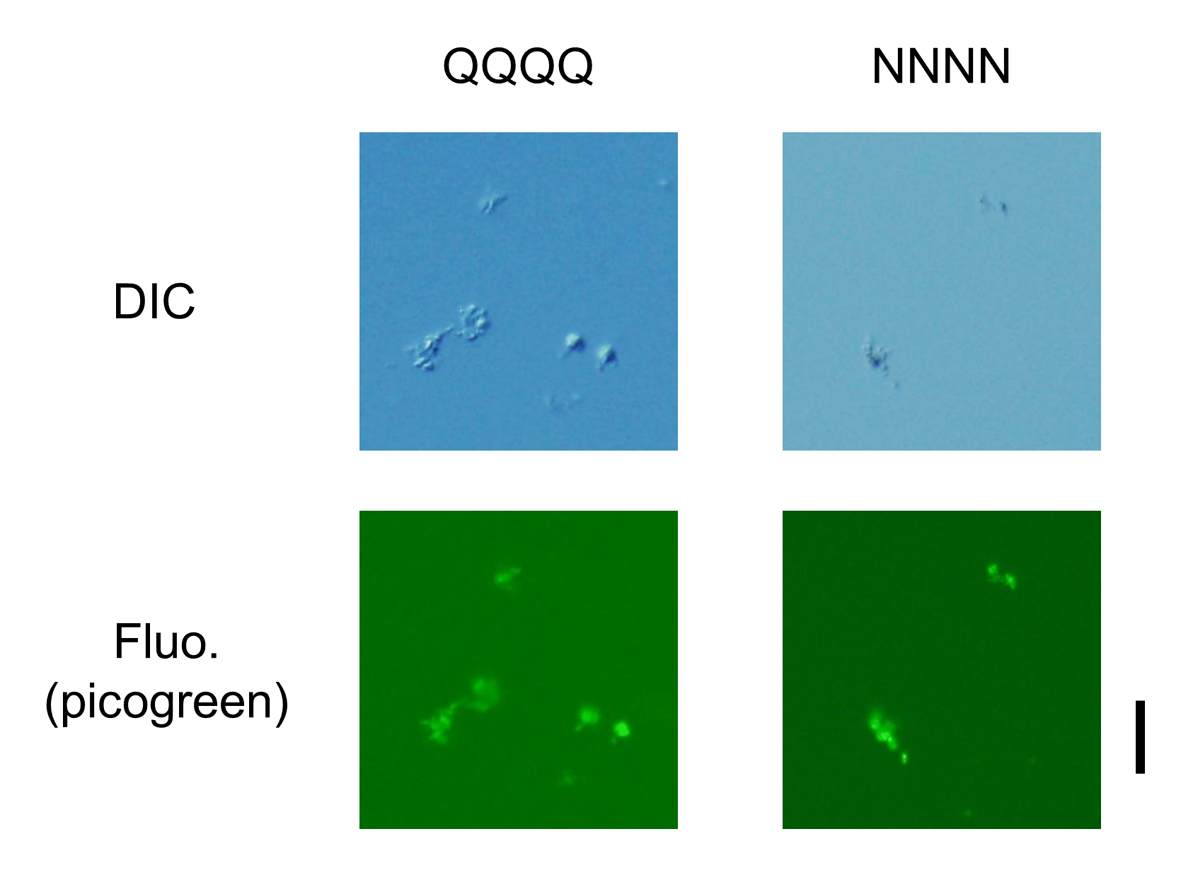


**Fig. S11.** DIC and fluorescence microscopic images of Q4 and N4 at high concentrations in the absence of FUS. The solution contained 30 mM Q4 or N4, 183 mM KCl, and 65 mg/mL Dextran. The fluorescence of amyloid sensitive Picogreen was detected. Scale bar denotes 10 µm.

**Supplemental text: Information regarding the charge of amino acid atoms.**

The charge of the amino acids modeled using quantum chemical calculations is described below in the Tripos mol2 format.

**Arg:**

@<TRIPOS>MOLECULE

ARG

27 26 1 0 0

SMALL

No Charge or Current Charge

@<TRIPOS>ATOM

1 N 3.5400 1.4200 0.0000 N3 1 ARG -0.611770

2 H1 4.2670 0.9590 0.5260 H 1 ARG 0.374033

3 H2 3.1440 0.7160 -0.6300 H 1 ARG 0.374033

4 H3 3.9580 2.1800 -0.5130 H 1 ARG 0.374033

5 CA 2.3790 1.8130 0.8840 CT 1 ARG 0.192343

6 HA 2.7760 2.0940 1.8480 HP 1 ARG 0.056317

7 CB 1.6380 2.9850 0.2120 CT 1 ARG -0.259857

8 HB1 2.3820 3.7250 -0.0710 HC 1 ARG 0.095967

9 HB2 1.1930 2.6260 -0.7110 HC 1 ARG 0.095967

10 CG 0.5800 3.7140 1.0810 CT 1 ARG 0.054325

11 HG1 0.7530 3.5210 2.1340 HC 1 ARG 0.038065

12 HG2 0.7130 4.7790 0.9480 HC 1 ARG 0.038065

13 CD -0.8890 3.4180 0.7540 CT 1 ARG 0.117976

14 HD1 -1.5120 4.1300 1.2830 H1 1 ARG 0.043674

15 HD2 -1.0670 3.5420 -0.3080 H1 1 ARG 0.043674

16 NE -1.2280 2.0600 1.1580 N2 1 ARG -0.576932

17 HE -0.5740 1.6110 1.7780 H 1 ARG 0.342767

18 CZ -2.0490 1.2200 0.5600 CA 1 ARG 0.941863

19 NH2 -2.9940 1.6550 -0.2720 N2 1 ARG -0.959313

20 HH3 -3.5980 1.0140 -0.7380 H 1 ARG 0.459605

21 HH4 -3.2750 2.6100 -0.2640 H 1 ARG 0.459605

22 NH1 -1.9110 -0.0730 0.8010 N2 1 ARG -0.959313

23 HH1 -1.0650 -0.3950 1.2390 H 1 ARG 0.459605

24 HH2 -2.5550 -0.7450 0.4490 H 1 ARG 0.459605

25 C 1.5180 0.5180 0.9680 C 1 ARG 0.770041

26 O 1.6450 -0.2250 0.0050 O2 1 ARG -0.712189

27 OXT 0.7780 0.4370 1.9500 O2 1 ARG -0.712189

@<TRIPOS>BOND

1 2 1 1

2 3 1 1

3 4 1 1

4 5 1 1

5 6 5 1

6 7 5 1

7 8 7 1

8 9 7 1

9 10 7 1

10 11 10 1

11 12 10 1

12 13 10 1

13 14 13 1

14 15 13 1

15 16 13 1

16 17 16 1

17 18 16 2

18 19 18 1

19 20 19 1

20 21 19 1

21 22 18 1

22 23 22 1

23 24 22 1

24 25 5 1

25 26 25 1

26 27 25 1

@<TRIPOS>SUBSTRUCTURE

1 ARG 1 TEMP 0 **** **** 0 ROOT

**Gly:**

@<TRIPOS>MOLECULE

GLY

10 9 1 0 0

SMALL

No Charge or Current Charge

@<TRIPOS>ATOM

1 N1 3.5400 1.4200 0.0000 N3 1 GLY -0.487586

2 H1 4.1050 2.3340 0.0030 H 1 GLY 0.324087

3 H2 2.9560 1.3500 0.8140 H 1 GLY 0.324087

4 H3 2.9640 1.3530 -0.8200 H 1 GLY 0.324087

5 C1 4.7180 0.4880 0.0030 CT 1 GLY -0.042396

6 H4 4.6940 -0.1310 0.8860 HP 1 GLY 0.083854

7 H5 4.6950 -0.1360 -0.8760 HP 1 GLY 0.083854

8 C2 5.9700 1.4340 0.0010 C 1 GLY 0.851416

9 O2 5.6310 2.6350 0.0020 O2 1 GLY -0.730702

10 O1 7.0380 0.8800 0.0000 O2 1 GLY -0.730702

@<TRIPOS>BOND

1 1 2 1

2 1 3 1

3 1 4 1

4 1 5 1

5 5 6 1

6 5 7 1

7 5 8 1

8 8 9 1

9 8 10 1

@<TRIPOS>SUBSTRUCTURE

1 GLY 1 TEMP 0 **** **** 0 ROOT

**Lys:**

@<TRIPOS>MOLECULE

LYS

25 24 1 0 0

SMALL

No Charge or Current Charge

@<TRIPOS>ATOM

1 N 3.5400 1.4200 0.0000 N3 1 LYS -0.606886

2 H1 2.9020 0.7710 -0.4370 H 1 LYS 0.371535

3 H2 3.9060 0.9600 0.8330 H 1 LYS 0.371535

4 H3 3.0210 2.2490 0.2480 H 1 LYS 0.371535

5 CA 4.7500 1.6830 -0.8630 CT 1 LYS 0.250913

6 HA 4.4320 1.6990 -1.8940 H1 1 LYS 0.046956

7 CB 5.3840 3.0250 -0.4420 CT 1 LYS -0.221549

8 HB1 4.8210 3.8280 -0.9070 HC 1 LYS 0.082035

9 HB2 5.2750 3.1460 0.6330 HC 1 LYS 0.082035

10 CG 6.8770 3.1630 -0.8120 CT 1 LYS -0.004370

11 HG1 7.0550 2.6770 -1.7620 HC 1 LYS 0.050694

12 HG2 7.0860 4.2150 -0.9590 HC 1 LYS 0.050694

13 CD 7.8380 2.6220 0.2800 CT 1 LYS -0.057954

14 HD1 8.1870 3.4570 0.8750 HC 1 LYS 0.053687

15 HD2 7.3140 1.9710 0.9710 HC 1 LYS 0.053687

16 CE 9.0840 1.8840 -0.2320 CT 1 LYS 0.035789

17 HE1 9.3820 2.2200 -1.2150 HP 1 LYS 0.075606

18 HE2 9.9190 2.0160 0.4400 HP 1 LYS 0.075606

19 NZ 8.8490 0.4050 -0.3360 N3 1 LYS -0.263625

20 HZ1 9.6400 -0.0710 -0.7430 H 1 LYS 0.287970

21 HZ2 8.6810 -0.0040 0.5720 H 1 LYS 0.287970

22 HZ3 7.9930 0.1950 -0.9020 H 1 LYS 0.287970

23 C 5.7050 0.5060 -0.5970 C 1 LYS 0.722530

24 O 6.5030 0.2790 -1.5130 O2 1 LYS -0.702181

25 OXT 5.6280 0.0140 0.5210 O2 1 LYS -0.702181

@<TRIPOS>BOND

1 2 1 1

2 3 1 1

3 4 1 1

4 5 1 1

5 6 5 1

6 7 5 1

7 8 7 1

8 9 7 1

9 10 7 1

10 11 10 1

11 12 10 1

12 13 5 1

13 14 13 1

14 15 13 1

15 16 13 1

16 17 16 1

17 18 16 1

18 19 16 1

19 20 19 1

20 21 19 1

21 22 19 1

22 23 5 1

23 24 23 1

24 25 23 1

@<TRIPOS>SUBSTRUCTURE

1 LYS 1 TEMP 0 **** **** 0 ROOT

**Phe:**

@<TRIPOS>MOLECULE

PHE

23 23 1 0 0

SMALL

No Charge or Current Charge

@<TRIPOS>ATOM

1 N -2.4750 1.6130 -0.3290 N3 1 PHE -0.684514

2 H1 -2.2700 2.3160 -1.0170 H 1 PHE 0.357221

3 H2 -2.6100 2.0620 0.5620 H 1 PHE 0.357221

4 H3 -3.3370 1.0580 -0.5480 H 1 PHE 0.357221

5 CA -1.4720 0.4840 -0.2570 CT 1 PHE 0.323339

6 HA -1.0240 0.4310 -1.2400 HP 1 PHE 0.025340

7 CB -0.3990 0.7210 0.8190 CT 1 PHE -0.092398

8 HB2 -0.3730 1.7760 1.0790 HC 1 PHE 0.042934

9 HB3 -0.6830 0.1760 1.7110 HC 1 PHE 0.042934

10 CG 0.9990 0.3120 0.3890 CA 1 PHE 0.043353

11 CD1 1.3210 -1.0270 0.1840 CA 1 PHE -0.201583

12 HD1 0.5630 -1.7750 0.3270 HA 1 PHE 0.166230

13 CE1 2.5990 -1.3830 -0.2100 CA 1 PHE -0.139827

14 HE1 2.8350 -2.4210 -0.3650 HA 1 PHE 0.138293

15 CZ 3.5730 -0.4140 -0.4040 CA 1 PHE -0.122231

16 HZ 4.5650 -0.6970 -0.7080 HA 1 PHE 0.130464

17 CE2 3.2600 0.9180 -0.2010 CA 1 PHE -0.139827

18 HE2 4.0080 1.6780 -0.3450 HA 1 PHE 0.138293

19 CD2 1.9790 1.2760 0.1930 CA 1 PHE -0.201583

20 HD2 1.7500 2.3160 0.3550 HA 1 PHE 0.166230

21 C -2.3780 -0.7890 -0.0620 C 1 PHE 0.655266

22 O -1.8090 -1.7840 0.3240 O2 1 PHE -0.681189

23 OXT -3.5650 -0.5660 -0.3530 O2 1 PHE -0.681189

@<TRIPOS>BOND

1 1 2 1

2 1 3 1

3 1 4 1

4 1 5 1

5 5 6 1

6 5 7 1

7 5 21 1

8 7 8 1

9 7 9 1

10 7 10 1

11 10 11 1

12 10 19 2

13 11 12 1

14 11 13 2

15 13 14 1

16 13 15 1

17 15 16 1

18 15 17 2

19 17 18 1

20 17 19 1

21 19 20 1

22 21 22 1

23 21 23 1

@<TRIPOS>SUBSTRUCTURE

1 PHE 1 TEMP 0 **** **** 0 ROOT

**Tyr:**

@<TRIPOS>MOLECULE

TYR

24 24 1 0 0

SMALL

No Charge or Current Charge

@<TRIPOS>ATOM

1 N -3.0060 1.4980 -0.3880 N3 1 TYR -0.594139

2 H1 -2.8410 2.2180 -1.0690 H 1 TYR 0.338577

3 H2 -3.2050 1.9290 0.4990 H 1 TYR 0.338577

4 H3 -3.8110 0.8740 -0.6340 H 1 TYR 0.338577

5 CA -1.9110 0.4590 -0.2860 CT 1 TYR 0.228627

6 HA -1.4340 0.4440 -1.2560 HP 1 TYR 0.041362

7 CB -0.8920 0.7960 0.8160 CT 1 TYR -0.066563

8 HB2 -0.9270 1.8640 1.0200 HC 1 TYR 0.039314

9 HB3 -1.1900 0.2870 1.7250 HC 1 TYR 0.039314

10 CG 0.5400 0.4410 0.4620 CA 1 TYR -0.008434

11 CD1 0.9580 -0.8820 0.3650 CA 1 TYR -0.164105

12 HD1 0.2470 -1.6690 0.5280 HA 1 TYR 0.183054

13 CE1 2.2680 -1.1890 0.0430 CA 1 TYR -0.347142

14 HE1 2.5730 -2.2200 -0.0290 HA 1 TYR 0.185577

15 CZ 3.1880 -0.1770 -0.1860 CA 1 TYR 0.474831

16 OH 4.4790 -0.4240 -0.5030 OH 1 TYR -0.618585

17 HH 4.6410 -1.3560 -0.5330 HO 1 TYR 0.422977

18 CE2 2.7900 1.1470 -0.0930 CA 1 TYR -0.347142

19 HE2 3.5090 1.9250 -0.2690 HA 1 TYR 0.185577

20 CD2 1.4770 1.4410 0.2290 CA 1 TYR -0.164105

21 HD2 1.1860 2.4750 0.3050 HA 1 TYR 0.183054

22 C -2.7100 -0.8860 -0.1060 C 1 TYR 0.703978

23 O -2.0710 -1.8260 0.3100 O2 1 TYR -0.696592

24 OXT -3.9010 -0.7700 -0.4360 O2 1 TYR -0.696592

@<TRIPOS>BOND

1 1 2 1

2 1 3 1

3 1 4 1

4 1 5 1

5 5 6 1

6 5 7 1

7 5 22 1

8 7 8 1

9 7 9 1

10 7 10 1

11 10 11 1

12 10 20 2

13 11 12 1

14 11 13 2

15 13 14 1

16 13 15 1

17 15 16 1

18 15 18 2

19 16 17 1

20 18 19 1

21 18 20 1

22 20 21 1

23 22 23 1

24 22 24 1

@<TRIPOS>SUBSTRUCTURE

1 TYR 1 TEMP 0 **** **** 0 ROOT
